# Overtraining Syndrome as a Complex Systems Phenomenon

**DOI:** 10.3389/fnetp.2021.794392

**Published:** 2022-01-18

**Authors:** Lawrence E. Armstrong, Michael F. Bergeron, Elaine C. Lee, James E. Mershon, Elizabeth M. Armstrong

**Affiliations:** ^1^ Human Performance Laboratory, University of Connecticut, Storrs, CT, United States; ^2^ Sport Sciences and Medicine and Performance Health, WTA Women’s Tennis Association, St. Petersburg, FL, United States; ^3^ Department of Energy and Renewables, Heriot-Watt University, Stromness, United Kingdom; ^4^ Riverside Behavioral Health Center, Hampton, VA, United States

**Keywords:** stress, exercise, hypothalamic-pituitary-adrenal axis, network, overreaching, metabolism, genome

## Abstract

The phenomenon of reduced athletic performance following sustained, intense training (Overtraining Syndrome, and OTS) was first recognized more than 90 years ago. Although hundreds of scientific publications have focused on OTS, a definitive diagnosis, reliable biomarkers, and effective treatments remain unknown. The present review considers existing models of OTS, acknowledges the individualized and sport-specific nature of signs/symptoms, describes potential interacting predisposing factors, and proposes that OTS will be most effectively characterized and evaluated via the underlying complex biological systems. Complex systems in nature are not aptly characterized or successfully analyzed using the classic scientific method (i.e., simplifying complex problems into single variables in a search for cause-and-effect) because they result from myriad (often non-linear) concomitant interactions of multiple determinants. Thus, this review 1) proposes that OTS be viewed from the perspectives of complex systems and network physiology, 2) advocates for and recommends that techniques such as trans-omic analyses and machine learning be widely employed, and 3) proposes evidence-based areas for future OTS investigations, including concomitant multi-domain analyses incorporating brain neural networks, dysfunction of hypothalamic-pituitary-adrenal responses to training stress, the intestinal microbiota, immune factors, and low energy availability. Such an inclusive and modern approach will measurably help in prevention and management of OTS.

## Introduction

All competitive athletes embrace daily rigorous physical training with the common goal of realizing their own athletic personal best and highest ambitions of sport success. In pursuit of performance excellence, the desired positive physiological adaptations are best achieved when the total workload, variations in activities, and intensity of exercise are appropriate and progressively introduced (i.e., considering fitness, prior training, health history, age, and hereditary potential), while complemented with regular and sufficient restorative rest. In contrast, a training regimen that is excessive, unduly straining, and without recurrent adequate recovery may provoke a range of maladaptations, resulting in stagnant or worsening exercise performance, undesirable mood/behavioral changes, and a greater risk of injury and/or illness (Kuipers and Keizer, 1988; Kraemer and Ratamess, 2005; Meeusen et al., 2013; Cadegiani and Kater, 2019a). This chronic, dysfunctional, maladapted state has been given various names (e.g., staleness, underperformance syndrome, under-recovery) since it was first recognized. Presently, the term overtraining syndrome (OTS) is widely recognized and accepted (Meeusen et al., 2013).

The prevalence of OTS is alleged to be highest in endurance sports requiring high volume and recurrent intense training such as swimming, triathlon, road cycling, and rowing (MacKinnon, 2000). High performance athletes in these sports frequently train for 4–6 h each day, 6 days per week, for several months without sufficient (if any) scheduled days of appropriate recovery. Accordingly, it is not surprising that 64% of female and 66% of male elite distance runners reported experiencing staleness at some point during their competitive careers (Morgan et al., 1987).

Specific contributing factors, signs/symptoms, and resulting maladaptive mechanism of OTS are variable and unclear. Thus, diagnosis and intervention to address OTS remain challenging for coaches, physicians, and physiologists. Too often, a coach or other member of the athlete support team (including parents/guardians) navigate an inefficient process to explain and remedy an undue performance plateau or decline. Clinical manifestations (e.g., generalized fatigue, insomnia, change of appetite, irritability, loss of motivation, and lack of concentration) may be helpful in identifying OTS. Yet, without an established effective and reliable therapy, even with an accurate diagnosis, athletes must often simply cease training to avoid long-term adverse impacts on their health and sport career (Meeusen et al., 2013). Typically, there is also no standard guidance on the recovery process and determining readiness for return-to-play. Accordingly, there has been considerable uncertainty and ineffectiveness in practical prevention strategies, sensitive early detection, and timely clinical and sport-specific management of those affected by OTS.

Supplementary Table S1 summarizes OTS publications in sport, medical, and applied exercise physiology journals between the years 1923–2018 and illustrates the slow evolution of relevant information and meaningful inherent concepts. Early anecdotal and case reports focused narrowly on signs and symptoms, whereas systematic studies, field research, and laboratory testing of overtrained versus asymptomatic athletes appeared only after 1985. Notably, before the year 2000, few of these publications proposed a hypothetical mechanism for OTS. However, casually employing the terms “syndrome” to address co-occurring clinical symptoms and “multi-factorial” to describe the general nature of OTS speak to the gaps and challenges in understanding OTS. Accordingly, we propose a complex systems approach to OTS that considers the co-occurrence and breadth of multi-factorial predisposing risk factors and clinical manifestations. This scheme also respects the extent and depth of high-order interactions among these contributing factors and resulting outcomes that characterize a dynamic and highly variable clinical condition within and among those affected. Our aim is that applying this more inclusive and modern approach will measurably help in revealing real-world practical prevention measures and establish more effective and individualized strategies for managing OTS.

## Prevailing Models of OTS

**FIGURE 1:**
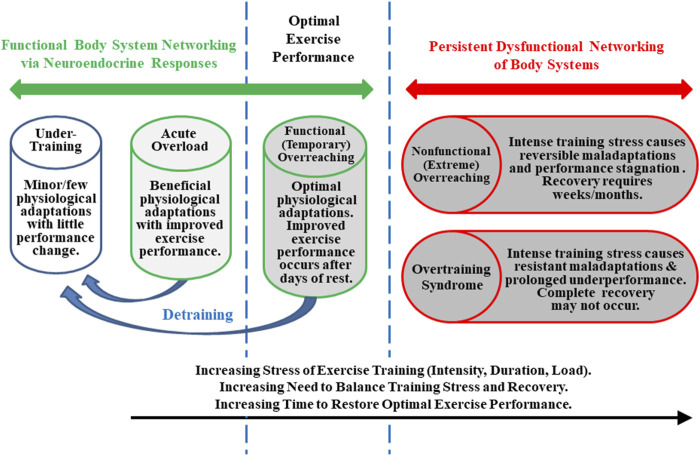
Training states that an athlete may experience throughout a competitive season. Although states and transitions are difficult to define and identify specifically, this figure is based on well-established concepts (Kuipers and Keizer, 1988; Fry and Kraemer, 1997; Kreider et al., 1998a; Armstrong and VanHeest, 2002; Meeusen et al., 2013).

To clarify its nature, investigators attempted to define OTS and theoretically distinguished it from overreaching (Figure 1), beginning in the mid-1980s (Kuipers and Keizer, 1988; Morgan et al., 1988; Fleck and Kraemer, 1982). OTS accordingly was defined as an accumulation of training and/or non-training stress and consequent strain which results in long-term athletic performance decrements that require several weeks or months to resolve. Estimates suggested that, at any given time, between 7 and 20% of athletes in all sports exhibit signs and/or symptoms of OTS (Morgan et al., 1987; Morgan et al., 1988; Hooper et al., 1993; Raglin and Morgan, 1994). By the mid-1990s, the term overreaching also had been popularized (Fry and Kraemer, 1997; Snyder et al., 1993). This purported (though somewhat arguably) discernably distinctive state was instead defined as an accumulation of training and/or non-training stress which results in short-term athletic performance deterioration that necessitates days to months of reduced training and recovery to subside (Halson and Jeukendrup, 2004; Urhausen and Kindermann, 2002; Kreider et al., 1998a). Although both conditions involved a decline of physical performance, the time required to restore performance (albeit indefinite and unpredictable) was the accepted critical difference between OTS and overreaching, not the degree of performance impairment or the extent and severity of signs and/or symptoms (Meeusen et al., 2013). Further, as illustrated in Figure 1, some authors (Armstrong and VanHeest, 2002; Urhausen and Kindermann, 2002; Halson and Jeukendrup, 2004; Meeusen et al., 2013) subdivided overreaching into two categories: functional (short-term) overreaching and nonfunctional (long-term) overreaching. The former involved desirable physiological adaptations (i.e., following intensified training with rest/recovery lasting several days) that enabled improved performance. The latter entailed extremely intensified training and physiological maladaptations (i.e., of critical organs, body systems, and/or metabolism) that prompted degraded exercise performance. Although some authors currently propose that nonfunctional overreaching and OTS exist on a single continuum that evolves with increasing stress, this may be an oversimplification (Meeusen et al., 2013). This is especially evident, given the diversity and nonlinearity of the numerous physiological, psychological, and physical interactions, presentation of clinical and performance manifestations, and changing time course of OTS among individuals. Moreover, various aspects of improved athletic conditioning/performance sometimes overlap with early and not readily apparent elements of undue physiological and/or psychological strain that begin to emerge with onset of OTS (Walsh, 1980; Halson and Jeukendrup, 2004).

A second OTS paradigm proposed two theoretical categories. In each, a different part of the autonomic nervous system predominates—sympathetic or parasympathetic (Fry et al., 1994; van Borselen, 1992; Lehman et al., 1993; Fry and Kraemer, 1997). Interestingly, the sympathetic form of this OTS model involved differentiating signs and symptoms including restlessness, excitation, insomnia, irritability, as well as increased heart rate, and blood pressure. The contrasting parasympathetic form was characterized by inhibition, fatigue, depression, apathy, and low resting heart rate (Lehmann et al., 1998). Sympathetic OTS more commonly affected individuals in anaerobic sports such as sprinting, jumping and throwing, while the more prevalent parasympathetic OTS was more often observed in highly trained endurance athletes involved in long-distance running, swimming, and road cycling (Lehman et al., 1993; Froelich and Heil, 1995). Further complicating any practical distinction in this paradigm, OTS signs, symptoms, performance decrements, and physiological responses among endurance athletes may or may not be analogous to those of resistance-trained athletes in anaerobic sports (Fry et al., 1994; Häkkinen et al., 1989; Kraemer et al., 1989; Fry et al., 1991). Moreover, the biochemical and clinical features of the sympathetic and parasympathetic forms of OTS (Lehman et al., 1993) have not been delineated clearly. Thus, this model has not been helpful in clarifying the etiology or principal mechanism(s) involved in OTS.

A third approach to elucidating the nature of OTS involved a theoretical comparison of training program variables, specifically volume versus intensity (Lehman et al., 1993). This concept dovetails with the dichotomy featured in the autonomic nervous system paradigm because the sympathetic form of OTS is supposedly associated with excessive training intensity and the parasympathetic form of OTS results primarily from excessive training volume. Defining OTS based on volume and intensity is, however, notably challenging given that numerous sports involve routinely changing disproportionate contributions of volume and intensity during training. The resulting impacts are further complicated by individual variable responses (Fry et al., 2005). Thus, in myriad sports, it is not realistically feasible to isolate volume vs intensity over variable and overlapping periods and phases of training to correctly predict risk or classify individuals in one of these two OTS categories.

Multiple other hypotheses have also been advanced to describe the nature and contributing factors of OTS (e.g., glycogen depletion, central fatigue, neuromuscular control, tissue trauma, immune system dysregulation, and glutamine depletion) (Fry et al., 2005; Carter et al., 2014). None of these proposed mechanisms sufficiently or reliably characterizes OTS in all sports. In practice, these and other OTS profile characteristics are not exclusive to OTS and moreover, if involved, the relationships are higher-order, complex, and consequently not consistent or thus explainable in simple linear terms. Thus, not surprisingly, no consistent biomarker or reliable laboratory test related to the above or other metrics has been identified and validated. The fact remains that 1) OTS is extremely difficult to elicit in controlled studies (Fry and Kraemer, 1997; Kraemer et al., 1998), 2) participants may not have reached a true state of OTS during experimental observations, despite intense and extensive physical training (Häkkinen et al., 1989; Meeusen et al., 2013), and 3) the clinical features of OTS differ from one individual to the next, usually are nonspecific, and may be anecdotal (Kuipers and Keizer, 1988; Morgan et al., 1988; Uusitalo et al., 1998; Eichner, 1995).

### Neuroendocrine Involvement in OTS

**FIGURE 2:**
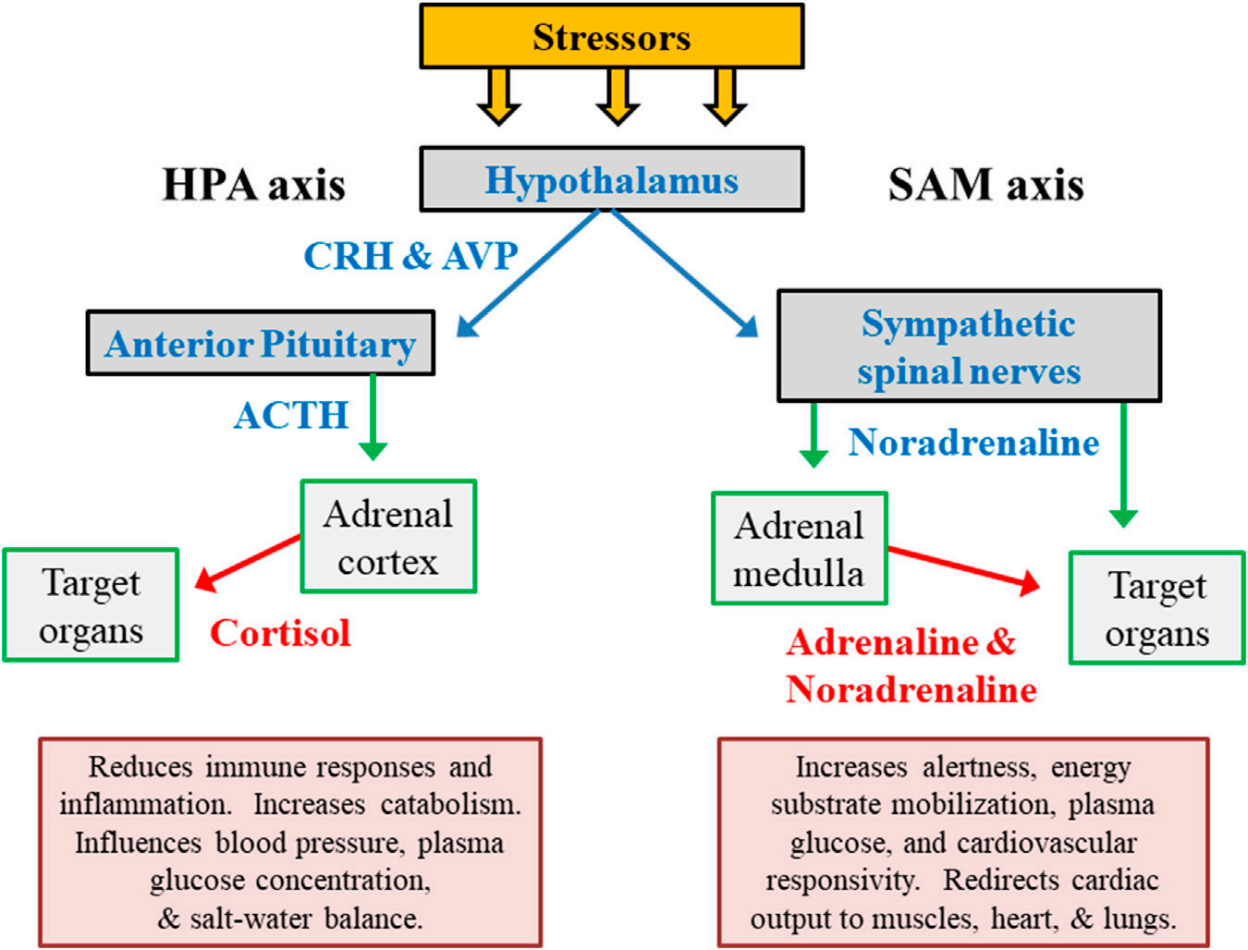
The primary hormone systems that respond to external and internal stress. The hypothalamic-pituitary-adrenocortical (HPA) axis represents releasing factors, produced by the hypothalamus (CRH in conjunction with AVP) and pituitary gland (ACTH), which lead to responses within the adrenal cortex and other peripheral organs/tissues. The sympathetic-adrenal medullary (SAM) axis represents the sympathetic branch of the autonomic nervous system. Abbreviations: ACTH, adrenocorticotropic hormone; CRH, corticotropin-releasing hormone; AVP, arginine vasopressin.

The hypothalamic-pituitary-adrenal (HPA) axis and the autonomic nervous system (ANS) are the primary regulators of human responses to stress (Figure 2). The immediate response to acute exercise stress, for example, is dominated by rapid ANS activation including its sympathetic-adrenal medullary (SAM) axis and sympathetic spinal nerve branches (Terjung, 1979). The adrenal release of cortisol peaks with a time lag of approximately 15 min after the encounter of a stressor. Complimentary feedback regulation is relatively slow, and cessation of the neuroendocrine stress response trails significantly behind the ANS (Deussing and Chen, 2018). In response to a variety of stressors (e.g., exercise, cold environment, hypoglycemia), a close association has been observed between HPA and SAM outputs (Goldstein and Kopin, 2008).

The primary hormonal products of the HPA and SAM axes (cortisol, adrenaline/epinephrine, noradrenaline/norepinephrine) temporarily reduce immune responses and inflammation, increase mental alertness, redistribute metabolic fuels, and modify cardiovascular responses (Figure 2). However, chronic stress-related adaptations (e.g., of neurotransmitter release, receptor sensitivity, receptor binding) in higher brain centers have a more enduring influence on numerous other psychological and physiological functions. This is because of the integration of numerous stressors by the hypothalamus and broader systemic impact (Lehmann et al., 1993; Lachuer et al., 1994).

OTS has been classically conceived by physiologists as an unintended outcome of chronic stress. Supplementary Table S2 presents summaries of publications spanning more than three decades, which focused on the neuroendocrine responses of athletes with OTS, with the majority of these being review articles. The fewer systematic laboratory and field studies of overtraining involved resistance training, running, swimming, and road cycling. Although the aggregate of these authors viewed neuroendocrine dysfunction as part of the etiology of OTS, this extensive body of research has failed to achieve widespread consensus on a profile of valid determinants or accepted practical and effective preventative measures.

**FIGURE 3:**
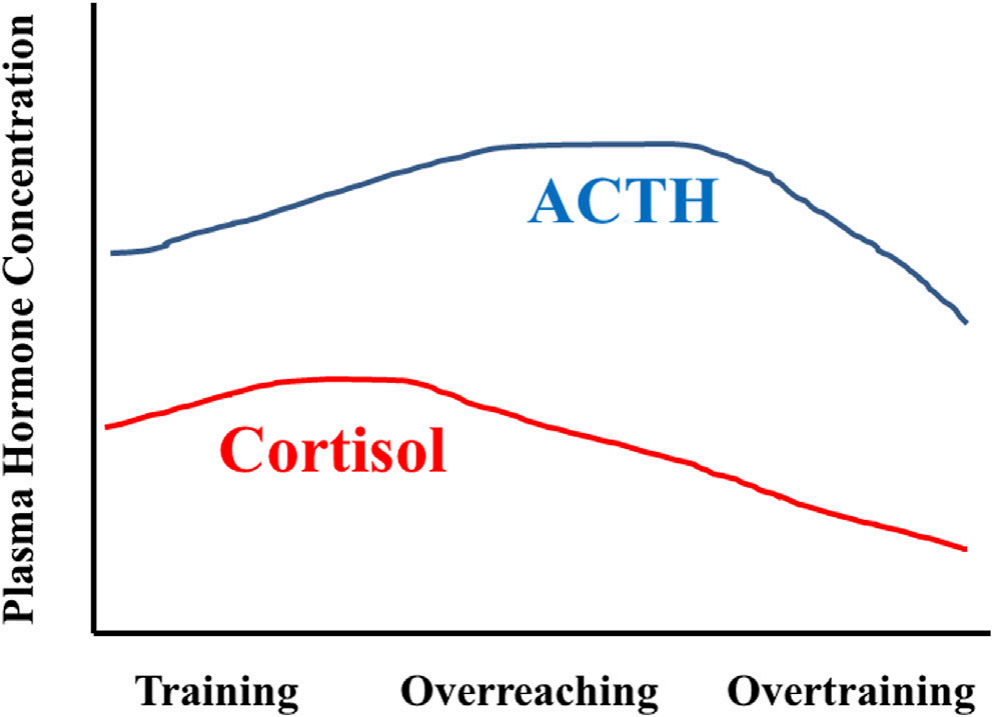
Idealized representation of HPA axis responses across months of training, as envisioned by Steinacker and colleagues in 2004 (Steinacker et al., 2004). During training with positive adaptations, ACTH and cortisol levels increase in response to the stress of training. During overreaching, the cortisol response is blunted whereas the ACTH response is augmented. Overtraining is characterized by decreased ACTH and cortisol responses. Figure modified and redrawn from (Steinacker et al., 2004). The original data sources were (Lehmann et al., 1993; Snyder, 1995; Wittert, 1996; Urhausen et al., 1998; Lehmann et al., 1999; Steinacker et al., 2004).

An early paradigm of a multi-phasic OTS distinguishes acute and chronic responses to training, with the emphasis on chronic training and combined life stress effects on neuroendocrine dysregulation. In this model, metabolic demands and strain, combined with exercise-induced peripheral muscle damage, are primary acute training responses, whereas changes in central nervous system (CNS) regulatory functions are more prominent with chronic training adaptations (Lehman et al., 1993; Steinacker et al., 2004). This model considers reduced exercise performance as a cumulative response to chronic inadequacy of post-exercise recovery time and ongoing non-exercise life stressors (Lehmann et al., 1993; Lehmann et al., 1997). Prolonged exposure to repeated, high-intensity training (without regular sufficient recovery) could drive decreased sensitivity of adrenal glands and HPA-axis function, manifesting as decreased plasma cortisol concentration (Figure 3). It was further argued that similar responses also might occur in all hypothalamic-pituitary-peripheral hormone axes (e.g., SAM). However, chronic exposure to undue stress (e.g., excessive increase in training load) prompts a range of varying CNS responses (i.e., unchanged, increased, or decreased) (MacKinnon, 2000; Meeusen et al., 2013). Thus, the current data suggest that chronic stress load (exercise and non-exercise) is not the exclusive determinant of neuroendocrine dysfunction in this OTS scenario.

Additional data shed light to potential contributions of other influences, such as would be evident from detraining, in neuroendocrine dysregulation associated with OTS. Comparison of athletes with OTS to healthy athletes and healthy-sedentary controls (Cadegiani and Kater, 2018a; Cadegiani and Kater, 2019b; Cadegiani and Sliva, 2020) revealed that athletes with OTS experienced deconditioning of useful training adaptations, including select endocrine benefits acquired by training (Cadegiani and Kater, 2018b). Loss of training benefits among athletes with OTS may not be inconsistent with previous multi-phasic OTS models. Rather, it provides opportunity for new interpretation based on the contributing complexity of influencing factors leading to OTS and consequent changes in neuroendocrine function. Direct comparison among different groups underscores the presence of key measurable and differentiating neuroendocrine aspects to OTS. Moreover, aligning inter-individual variability in biomarkers with dynamic, intra-individual circadian irregularity provides further potential useful perspective and insight to additional mechanisms and their novel roles in neuroendocrine changes with OTS.

Numerous models and experimental designs from studies that assessed neuroendocrine responses (Supplementary Table S2) and their resulting inferences have been criticized for a variety of significant reasons. First, athletes with OTS, healthy athletes, and sedentary non-athlete controls are known to exhibit different neuroendocrine responses (Cadegiani and Kater, 2019a; Cadegiani and Kater, 2019c), but these 3 groups have been directly compared in only a few previous OTS studies. Second, basal plasma stress hormone levels (i.e., cortisol, norepinephrine, and epinephrine) likely will not reliably distinguish athletes with OTS from healthy individuals (Cadegiani and Kater, 2017). Thus, measuring stress hormone levels during exercise may be a valid and more revealing approach (Meeusen et al., 2010; Meeusen et al., 2013). Third, few of the neuroendocrine assessments of athletes with OTS have involved validated and widely recognized standardized tests that are endorsed by professional endocrinology societies. These established assessments (e.g., insulin tolerance test, cosyntropin stimulation test, salivary cortisol rhythm test (Cadegiani and Kater, 2019c) allow valid comparisons with non-physically active controls and across studies to better clarify persistent neuroendocrine maladaptations due to OTS (Cadegiani and Sliva, 2020). Fourth, because valid test participant inclusion criteria were often not defined or applied rigorously, certain studies may have unknowingly evaluated athletes with nonfunctional overreaching and not OTS (Meeusen et al., 2013). Neuroendocrine data analysis in future OTS studies will be improved by addressing these concerns and applying methods that evaluate patterns of dynamic interaction across multiple determining factors.

## Characteristics and Methods of Complex Systems Analysis

Complex systems in nature have numerous integrated and interdependent functional components that generate variable outputs which cannot be characterized or consistently predicted based on discrete inputs alone (Table 1). Within individuals, these inherent properties invariably evolve across time and in proportionate corresponding response to relevant stressors. Anticipating, for example, the response and degree of influence by a complex system is challenging, given that the higher order supporting or constraining interactions among expected fundamental determinants (e.g., risk factors for illness or injury risk) are frequently not readily observed and thus not fully appreciated or remain unknown. Consequently, their direct or secondary relationship with the outcome is seemingly weak or considered non-existent (Freckleton and Pizzari, 2013). An effective (albeit, not trivial) means to infer complex system dynamics is to first map the interactions of potential predisposing factors. Secondly, identify predominant patterns of interaction and calculate the probability that each pattern will result in a specific clinical condition (i.e., create a risk profile). Finally, using the likely high-value features, develop and validate classification models that are sensitive to higher order relationships and interactions for predicting specific short-term and long-term effects (Schwartz, 2020).

**TABLE 1:** General characteristics of complex systems in nature, and specific characteristics of complex brain networks.

Complex systems in nature
• Many systems (i.e., medical, physiological, economic, and traffic flow patterns within cities) can only be properly characterized by multiple interdependent networks whose normal functions depend on one another Buldyrev et al. (2010)
• A complex task is one for which many factors must be considered to determine the outcome (i.e., product) of an action. The sources of complex tasks are complex systems (i.e., systems with interdependent parts). Interdependence means that a system’s behavior/mechanism cannot be determined by considering each of the parts and combining them. Instead, it is preferable to consider how the relationships between the parts affect the behavior of the whole Bar-Yam and Institute (2003)
• In complex (versus simple) systems, each outcome can be generated in multiple diverse ways Von Bertalanff. (1950), such that the statements X causes Y, and Z causes Y are not mutually incompatible
• Conceptual paradoxes also may coexist in complex systems (e.g., random and predictable, ordered and disordered, stable and adaptable, deterministic, and chaotic) Bar-Yam and Institute. (2003)
• Terminology: A network *node* is a point at which subsidiary parts originate or center (i.e., a point of connection); it has one or more physical links to other nodes. For example, a traffic intersection is a node because multiple roads connect at that point. A *module* in a network is a set of nodes that have strong interactions and a common function. Modules often are characterized by short metric distances between linked pairs of nodes. A network *link* is the connection between one node and another (i.e., coupling), for the purpose of transmitting and receiving information. The arrangement of elements (links, nodes, etc.) within a communication network is known as its *topology* Aderem. (2005), Sporns et al. (2004)
• Nodes and links in the human body often number in the thousands or millions. Such networks are complex because of their size and because of the behavior of individual nodes Sporns et al. (2004)
• Inputs to a complex system are not necessarily proportional to its output [50]. Large changes in one node/variable do not necessarily produce a large effect on the outcome. Conversely, small changes in one node/variable may produce large and unexpected effects on the outcome/product Holland, 1995; Coffey (1998); Higgins. (2003)
• Failure or dysfunction of one or several nodes in network A can lead to failure in network B, which may cause malfunction of additional nodes in network A. Such a cascade of failures may lead to the complete fragmentation of all or a majority of interconnected networks Buldyrev et al. (2010)
• The existence of modules can contribute to both robustness of the entire system by confining damage to separable parts, and to system evolution by rewiring modules. Further, modularity decreases the risk of failure of the system by preventing the spread of damage in one part of the network throughout the entire network (Aderem, 2005)
**Complex brain networks**
• Complex brain networks exhibit the characteristics of complex systems in nature (above)
• The study of complex networks offers new insights into both global and integrative aspects of brain function. For example, cerebral cortical areas are neither completely connected with each other nor randomly linked; their interconnections show a specific and intricate organization Sporns et al. (2004)
• Understanding the relationship between topology and the dynamic behaviors of complex networks in the human brain is challenging. Each node (or brain locus) administers a multicomponent system with its own regulatory mechanisms, the output of which varies across time. Further, specific neural links can be strengthened or weakened, and network topology can evolve Bashan et al. (2012)
• Modules exist in the human brain that are characterized by surprisingly short metric distances between linked pairs of nodes, within very large networks Sporns et al. (2004)
• Published observations of human sleep stage transitions identified a robust network of interactions between physiological systems, which remained stable during a given sleep stage. Changes of the sleep stage (i.e., and the accompanying physiological responses) led to complex network transitions within minutes. These involved (a) a structured reorganization of connectivity and topology throughout the entire network, and (b) formation of physiological subnetworks with different topologies and behaviors Bashan et al. (2012)

Previously published OTS stress studies are typically characterized by only a few measured time points and a limited number of physiological variables (e.g., the HPA and SAM hormones cortisol and norepinephrine, expression of a few genes). However, investigators increasingly have recognized that a single or even several outcome metrics, predisposing factors, or pathways of influence cannot sufficiently capture the complexity and holistic context of the body’s numerous simultaneous and interdependent responses to any stressor (Schwartz, 2020; MacDougall-Shackleton et al., 2019; Dickens and Romero, 2013; Breuner et al., 2012). The etiology of OTS is highly complex, to-date not adequately described, and presents with numerous/varied signs and symptoms that depend on the type and intensity of training and inherent profile characteristics of the affected individuals (Supplementary Table S1). Nonetheless, we consider the expert consensus statement, published jointly by the European College of Sport Science and the American College of Sports Medicine, to be the foremost current resource of information regarding OTS and nonfunctional overreaching (Meeusen et al., 2013). This comprehensive expert consensus recognizes that OTS represents a complex syndrome. Deliberately using the specific term *syndrome* acknowledges the multi-factorial etiology of OTS which, in turn, supports that excessive exercise training is not the sole causative factor (1). Moreover, this document also notably cites evidence that prolonged maladaptation of regulatory mechanisms occurs concomitantly in multiple neurochemical and endocrine systems. However, this adjectival clinical semantic and noted multi-system responses still underrepresent the higher-order interdependencies underlying the condition. Accordingly, a complex systems (Table 1) approach is required to fully appreciate, aptly analyze, and accurately reveal the intractable complexity of OTS.

### A Novel Complex Systems Model of OTS

That OTS develops when a disproportionate chronic imbalance exists between excessive training and rest/recovery is a message without a clearly defined etiology or consistent profile of underlying physiological mechanism(s). It is further likely that the distinct pattern and pace of OTS emergence, progress, and outcomes (i.e., end phenotypes) are uniquely determined by the individual’s inherent responses to the diverse and complex interactions of the contributing stressors. The possible permutations of stressors, genotype, cellular damage, adaptations, maladaptations, inadequate repair, maturation/aging, and environmental factors are effectively endless and beyond one’s practical ability to consider. Thus, a complex systems approach arguably offers the best path forward, as we seek to clarify and appreciate the nature and individualized signature of OTS. The following eight tenets are offered as evidence that OTS has the characteristics of a complex system and thus warrants this approach.

•Other than a decline of exercise performance, no single biomarker or host characteristic definitively distinguishes athletes with OTS from healthy athletes or validly predicts risk or the onset of OTS (Meeusen et al., 2013; Cadegiani and Kater, 2018a; Cadegiani and Kater, 2019b; Cadegiani and Kater, 2019c; Cadegiani and Sliva, 2020).
•The search for a single supporting physiological mechanism (e.g., impaired immune response) has been unproductive because OTS stems from unique interactions of the host genome combined with the select interplay of multiple risk factors and chronic stressors that lead to marked dysfunction (Meeusen et al., 2013; Bittencourt et al., 2016; Cadegiani and Kater, 2019b).
•Athletes respond to excessive training loads in highly individualized ways (MacKinnon, 2000), and the signs and symptoms of OTS (e.g., performance decline, psychological strain, immunological, biochemical responses) are diverse (Hackney and Lane, 2015).
•An athlete’s genome and training/workload history (i.e., recent and chronic physiological demands and adaptations across multiple years) influence the variable training intensity-volume threshold at which OTS begins to appear (i.e., initial evidence of an undue decline of performance, mood changes, and/or poor quality of sleep).
•Attempting to identify a single causative etiological factor for OTS (e.g., training intensity or volume, skeletal muscle microtrauma) has been ineffective because athletes dynamically adapt or maladapt each day to the varying stresses of training and competition (Figure 1 (MacKinnon, 2000)); accordingly, their physiological and mental responses are constantly in transition. For this reason, an athlete’s training and related health status can only be approximated at the time s/he is observed by a sports medicine physician, exercise physiologist, or coach (Meeusen et al., 2013).
•The duration of OTS is indefinite and its prognosis is undefined and uncertain.
•The endocrine (i.e., cortisol, adrenocorticotropic hormone, epinephrine, norepinephrine, insulin, glucagon, growth hormone, testosterone), nervous (i.e., sympathetic-parasympathetic balance), muscular (i.e., hypertrophy, fiber type), circulatory (i.e., cardiac output, vascular tone), and metabolic (i.e., glycolysis, Krebs cycle, beta-oxidation) responses are specific to the predominant activity performed (e.g., endurance versus resistance training stresses) (Hackney and Lane, 2015). Further complicating these numerous interactions, many sports and sport-specific training methods possess a combination of high-force and high-endurance characteristics (Hooper et al., 2020).
•During the development of OTS, non-exercise life stressors such as inadequate nutrition (i.e., total energy intake, percent carbohydrate), psychosocial difficulties (e.g., team, coach, and family), disrupted sleep, and occupational cognitive demands add to the physical and mental stresses of strenuous training and sport competition (Meeusen et al., 2013; Cadegiani and Kater, 2018a).

To summarize these tenets: highly individualized signs and symptoms are observed among athletes with OTS, physiological and mental states are constantly transitioning in response to daily training and competition stresses, the clinical diagnosis of OTS is by exclusion, no single biomarker is known, the predominant physiological mechanism has not been identified, the duration of OTS is indefinite and highly individually variable, and life stressors unrelated to exercise add to the stresses of excessive training and competition load and thus also affect OTS risk. In total, these characteristics underscore the fundamental and remarkable complexity of OTS and explain why reductionist scientific approaches (i.e., seeking discrete cause-and-effect) previously have been ineffective. Thus, the inherent challenges warrant support for a complex systems approach to evaluate, establish a new paradigm for, and reveal the underlying nature and signature of OTS.

To uncover and clarify the behavior of a complex system and more precisely differentiate the scenarios and contributing features that most influence and determine classification of linked effects, it is necessary to apply non-linear computation methods. These analytic strategies more closely parallel how complex systems can simultaneously facilitate and constrain affected outcomes in a non-linear way, where small changes in just a few determinants can lead to large and sometimes unexpected advantages or consequences. Accordingly, the behavior of a complex system can best be revealed by pursuit of prediction, not causality. Moreover, practical prevention is best achieved by clear and valid prediction. Thus, we recommend that statistical models be adopted in OTS studies to investigate dynamic interactions among many different predictors, with the final goal of identifying patterns/outcomes that have a high probability of occurring—that is, pose the greatest modifiable threat/risk to athletes.

**FIGURE 4:**
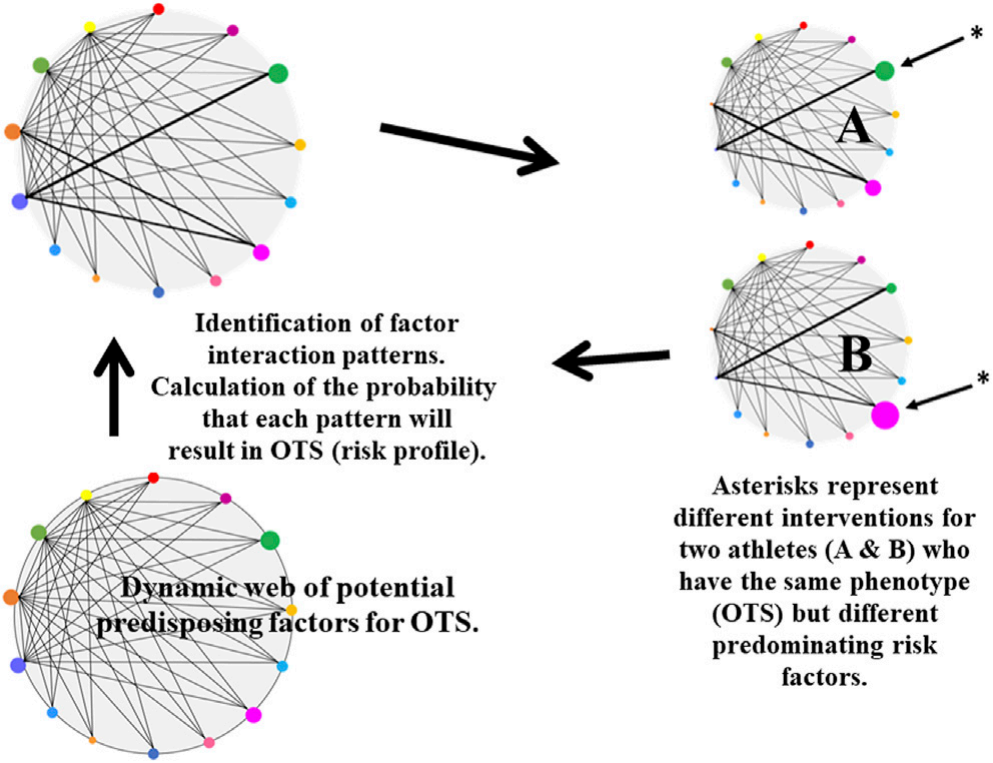
Proposed complex systems assessment of the Overtraining Syndrome (OTS). The classic scientific method of reductionist analysis is ineffective when studying OTS because multiple factors interact simultaneously. Potential predisposing factors for OTS (illustrated here as multicolored nodes) are linked to each other in a non-linear web (lower left quadrant). The nodes of this web may be host characteristics (e.g., hereditary capabilities, training adaptations) or may arise from the environment (e.g., training intensity/volume, nutrition, and non-exercise life stressors). A complex systems analytical approach explores and reveals the inherent interaction patterns of predisposing factors (upper left quadrant) in athletes who exhibit OTS. By calculating the probability that each interaction pattern will result in OTS, a risk profile is developed for each athlete (upper right quadrant); this profile is applied by modifying high-risk factors (e.g., reduced training load, increased rest/recovery, increased dietary carbohydrate). Varying node sizes and intensity of connecting lines between nodes represent variability in the impact of factors (nodes) and strength of interactions (bold lines). The cyclical nature of this method accommodates both positive adaptations (e.g., improved strength and endurance) and counterproductive maladaptations (e.g., OTS); it also implies that athletes should be reevaluated regularly throughout a training cycle or season to update the risk profile.

Figure 4 presents our proposed approach to the evaluation of OTS. A dynamic web of potential predisposing factors for OTS evolves as the body experiences recurring high volume-intensity training and other contributing stresses. The complex ongoing interactions of these factors eventually enable the formation of regularities which emerge as individualized characteristic patterns of positive adaptation or undesirable and counterproductive maladaptation (e.g., OTS). Instead of focusing on discrete relationships among individual components of this intricate biopsychosocial web, a complex systems analysis initially explores and reveals the inherent patterns of interaction among the wide array of potential interrelated predisposing factors. These patterns can then inform the appropriate categorical classification approach. Their selected utility in the model (based on probability, Figure 4) are thus integral to accurately predicting OTS. Including a wider range of high-probability patterns (interactions of predisposing factors) enables a greater degree of risk stratification precision, across more diverse profiles of individuals and groups of athletes. This could not be as practically applied and clearly revealed by focusing on selected discrete measure cause-and-effect. Within a group of athletes, for example, a few may exhibit the same phenotype (OTS) but possess different predominant risk factors. Thus, the benefits of employing high-performing models will be realized by 1) more readily enabling prompt individualized interventions for those at greatest risk or beginning to experience OTS signs and symptoms, and 2) reducing the time to achieve significant and meaningful athlete performance improvements.

Notably, Figure 4 generates uncertainties regarding current terminology and the very nature of OTS. The two athletes (A and B) are initially diagnosed/classified with the same phenotype (i.e., OTS) because their performance decreased following sustained intense training. However, a complex systems assessment (i.e., producing an individualized risk profile and signature) may reveal that different predisposing factors predominate in athletes A and B and thus different interventions are required. If a more clear delineation of multiple interactive determinants and a corresponding variable hierarchy of high-risk predisposing factors can be identified (Figure 4), it is then plausible that OTS has multiple subtypes (e.g., predominantly characterized by HPA axis dysfunction, low energy availability, or impaired immune function). Alternatively, the syndrome currently defined as OTS could represent multiple separate clinical conditions that require distinct specific interventions (i.e., athletes A and B with OTS in Figure 4 do not share an identical phenotype). These uncertainties are worthy of future research, as clarification would practically impact prevention strategies and clinical case management.

### OTS Research: New Analytical Methods and Technologies

Current approaches to improve predictive capabilities via complex systems analysis aim to identify stable interactions among predisposing factors by employing a variety of pattern recognition techniques. Contemporary examples of these techniques include Self-Organizing Feature Maps (based on unsupervised machine learning) that predict athletic talent, complex system analysis in weather forecasting, physiological and behavioral biometrics recognition using unsupervised and supervised machine learning, regression trees, random forests, and artificial neural networks in medicine and medical imaging, and agent-based modeling in epidemiology (Meeuwisse et al., 2007; Bittencourt et al., 2016; Ortiz et al., 2018). Iterative machine learning (statistical learning) techniques also have been applied to enhance predictive capability in the evaluation of numerous other complex systems. Notably, the power of these resulting practical and high-value predictive models could not have been emulated solely by basic traditional statistical methods (i.e., experimentally analyzing only single or few variables to confirm/refute cause-and-effect hypotheses) (Hastie et al., 2001; Andrés-Rodríguez et al., 2019). Machine learning approaches are also being increasingly utilized in sports medicine and other aspects of healthcare. For example, a comparison of 10 classification models developed with machine learning demonstrated practical application in predicting the resolution time of concussion-related symptoms that were incurred in high school sport participation (Bergeron et al., 2019). This modern classification approach was similarly effective in developing a 28-variable based model that exhibited 81% accuracy in discriminating severely ill patients with COVID-19 from those with only mild symptoms (Yao et al., 2020). And despite the inherent challenges in detecting mild dehydration, predictive models developed with machine learning performed well in stratification of hydration status prompted by autonomic nervous system responses to cognitive stress (Posada-Quintero et al., 2019). Whereas there was not a clear advantage in using machine learning over traditional statistics in these examples, it’s notable that more researchers are learning and applying these modern methods that are becoming more conventional. This will likely pay practical dividends with analyzing complex systems and datasets characterized by greater dimensionality and higher-order non-linear inter-relationships among variables, as with OTS.

A traditional statistical approach to studying OTS via its underlying complex biological systems was employed by Cadegiani and colleagues (Cadegiani and Kater, 2020). Their procedures began with 67 metabolic, hormonal, biochemical, anthropomorphic, and clinical variables, measured in 51 male athletes (14 with OTS, 25 healthy athletes with no signs or symptoms of OTS, 12 sedentary control subjects). Athletes with OTS were selected based on verified underperformance (e.g., coach’s assessment), persistent fatigue, >300 min/wk of moderate-to-vigorous intensity exercise, and continuous training for at least 6 months. Utilizing probability calculations, their results suggested that OTS resulted from training stress combined with unanticipated, non-exercise risk factors and behaviors, including insufficient caloric, protein or carbohydrate intake, poor sleep quality, and excessive cognitive effort outside of the training environment (Cadegiani and Kater, 2019b; Cadegiani and Kater, 2019c; Cadegiani and Sliva, 2020). Although the authors concluded that this statistical approach successfully distinguished athletes with OTS from healthy athletes and sedentary controls, it would have been interesting to determine whether a statistical learning approach might have provided greater predictive classification performance. As with classifying sport-related injury and other profiles of disease/illness risk, prevention strategies and clinical management of OTS would benefit from more precise discriminant stratification (beyond simple dichotomous classification) of individuals and potential effective interventions and therapies. With larger and higher-dimensional datasets, more robust machine learning models and application of deep learning ensemble methods are more likely to achieve this aim.

Physiologists, analyzing the human body as a complex system that responsively and promptly adapts to internal and external stressors, have employed novel statistical and mathematical tools to model selected inherent dynamic, nonlinear responses. For example, Ivanov and colleagues (Ivanov et al., 2014) successfully integrated physiological outcomes with innovative statistical techniques to identify an interactive network of 10 variables from six physiological systems (i.e., heart rate, respiration, electroencephalographic activity, chin muscle tone, leg and eye movements) during evolving stages of sleep. Changes of sleep stage led to complex network transitions within minutes, including formation of physiological subnetworks with different topologies and behaviors (Bashan et al., 2012). Relevant to the present review, similar approaches have been proposed for the assessment of training stress, overreaching, and OTS (Balagué et al., 2020).

Technological developments during the 1980s permitted potential determinants of specific diseases and cellular functions to be analyzed from a *Big Data* perspective—that is, concomitantly utilizing massive available data inputs from the entire relevant ecosystem. Automated DNA instrumentation enabled the sequencing of genomes and identification of individual polymorphisms, microarray analysis permitted global transcriptional profiling, and advances in mass spectrometry led to large-scale analyses of the primary derivatives and byproducts of protein synthesis and metabolism. Armed with terabytes of data generated by high-throughput laboratory capabilities, an exponential growth in the study of complex systems rapidly followed (Aderem, 2005). For the future study of OTS, these analytical methods will provide unparalleled insight and discovery that will reveal yet-to-be-established behaviors of complex human systems rather than simply accumulate data about them. We anticipate that these methods will lead to an enhanced capacity to describe key relationships, help in recognizing and appreciating the purported relevance of genotypes to function, and assist in practically anticipating and distinguishing presumed dynamic responses (Bar-Yam and Institute, 2003) and patterns among athletes with OTS, healthy athletes, and sedentary controls.

Fortunately, for today’s researchers, there are numerous no- or low-cost, popular, and readily accessible programming languages and dedicated platforms to support statistics, data science, and machine learning (e.g., Python/scikit-learn, R, and Weka). Moreover, rapidly evolving new technologies designed for direct user interface may prove to provide revealing novel insights that will reinforce and complement the evolution and practical utility of future innovative models applied to complex human systems. However, the demonstrated validation, reliability, integration, and practical application/value of the broad category of these consumer technologies composed of smart watches (integrated aggregation of metrics and bi-directional communication with the user and the cloud), strap/band/patch devices (direct collection and measurement of physiological metrics such as glucose, sodium, oxygen saturation), garments with embedded sensors, and task-based computer software/mobile applications will be essential before these technologies should be considered acceptable for local or remote screening, profiling, differentiating, tracking, and mitigating risk of OTS in athletes.

### Analysis of OTS Complexity Using Trans–omic Methods

**FIGURE 5:**
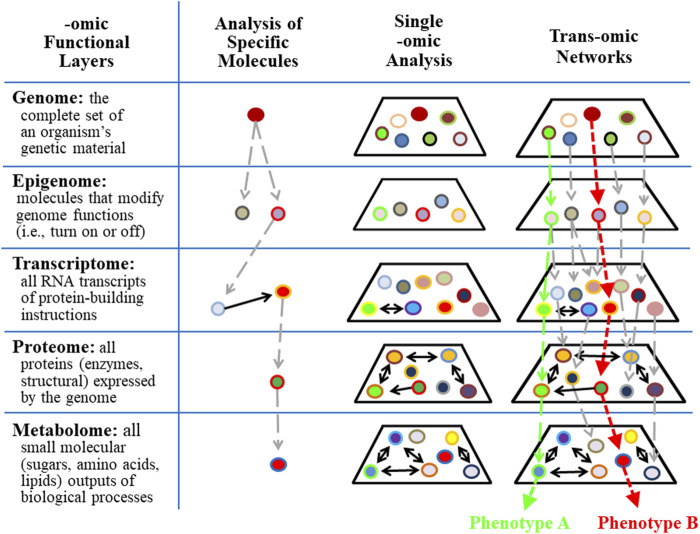
Trans-omics analyses improve the precision of detecting individual responses in complex systems. Single or panel biomarker analyses are limited in their ability to predict outcomes associated with complex systems that contain redundant, multi-functional variables. Trans-omics (integrative-omics) analysis permits a broad landscape view of thousands of contributing factors and patterns that lead to global outcomes (e.g., phenotype A vs. phenotype B) which otherwise would be undetectable by single biomarkers.

Biochemical pathways and networks have traditionally been identified by accumulating information about specific molecules. These analyses are represented in column 2 of Figure 5. Subsequently, specific levels of cellular complexity were defined and categorized according to the basic building blocks of the cell, including DNA, RNA, protein, and the products of metabolism. Each of these molecular hierarchical levels has internally consistent chemical properties and is named an “-omic” layer (e.g., genome, epigenome, transcriptome, proteome, and metabolome) (Ivanov et al., 2014). The addition of the suffix omic to a cellular term implies a comprehensive/global assessment of a set of molecules (Balagué et al., 2020). Five -omic levels are illustrated and defined in Figure 5 as horizontal rows. Complex biological organization and processes can be studied in terms of molecular constituents, within and across -omic levels.

The behaviors of single -omic layers and networks within each -omic layer have been identified (see column 3 in Figure 5) using state-of-the-art measurement technologies. The epigenome is particularly relevant to the study of OTS because epigenetic modification of molecular processes provides a response memory (i.e., of a previous encounter) that primes cells and tissues for future encounters with the same stress/stimulus. This memory is evidence that information is retained for later experiences and suggests neural plasticity and programmability (Sharples et al., 2016). For example, epigenetic modifications of gene expression following a single bout of exercise may involve restructuring skeletal muscle and tendons to better handle mechanical strain, or the responsive adaptations may involve altered metabolism that is specific to the intensity, duration, and mode of exercise (Saghiv and Sagiv, 2020). Accordingly, during prolonged periods of intense training, the adaptive responses of each exercise bout are integrated and interdependent (i.e., via numerous epigenetic modifications of gene expression). The result is gradually improving or degrading performance over time (Rasmussen et al., 2014) that is also subject to environmental influences other than excessive training, including diet, stress, infections, and disease (Alegría-Torres et al., 2011; Pareja-Galeano et al., 2014; Hackney and Lane, 2015; Widmann et al., 2019).

However, a single -omic level analysis alone does not directly identify interactions across multiple -omic levels. To overcome this information gap, an approach for reconstructing molecular networks by connecting multiple -omic data has been developed and named trans-omics (Yugi et al., 2016) or integrative-omics. Cellular functions are orchestrated by global networks that transect multiple -omic layers and these chains of direct mechanistic molecular interactions are the essence of trans-omic analyses. Two trans-omic pathways are illustrated in column 4 of Figure 5 as vertical green and red dashed arrows. Each results in a unique phenotype. Notably, many avenues of higher-level, more integrative -omics fields may be added to complement this model that would extend the functional schematic and representative impact (e.g., organomics, physiomics, and environmentalomics). Examining any aspect of this trans-omic system, such as in translating cellular and tissue level -omics analyses to organismal function while interacting with the environment, is more aptly pursued with deep (machine) learning and artificial neural networks that more suitably represent the inherent complex network physiology.

Investigators who specialize in human stress responses recognize that measuring a single variable or considering only one -omic layer is not sufficient to fully characterize or interpret the intrinsic complexity and context of the stimuli-response pathway(s). This underscores why a universal single endocrine profile has not been identified and validated for chronic stress (Schwartz, 2020; MacDougall-Shackleton et al., 2019; Dickens and Romero, 2013; Breuner et al., 2012), a perspective that fittingly applies to OTS. Accordingly, we propose trans-omic research to fully characterize the nature and the complex web of interrelated predisposing factors of OTS (lower left quadrant of Figure 4). Such an exploratory approach should include rapid quantification of molecular-level phenomena that spans the entire cell or tissue, in contrast to more traditional molecular-scale measurements (Robinson and Nielsen, 2016).

Specific to OTS research, trans-omic methods have great potential to 1) indicate if an individual or sub-population is (and/or has been) under stress, 2) differentiate contributing stressor types, 3) distinguish patterns and phases of an integrated stress response, 4) discern how acute and chronic stressors may impact health and performance differently, and 5) identify alternative genetic mechanisms for a given response to a stressor (Schwartz, 2020). Also with OTS, application of trans-omic data may well enhance our understanding of the molecular dynamics underlying its pathophysiology, identify longitudinal effects during the emergence of OTS (Ritchie et al., 2015), and lead to novel validated strategies for early detection, prevention, and treatment (Civelek and Lusis, 2014; Sun and Hu, 2016; Angione, 2019).

## Potential Areas for Future Research

In the following paragraphs, we propose specific areas for future research that are viable in clarifying the nature and complex etiology of OTS. Based on evidence from the open scientific/clinical literature, these areas are likely candidates for inclusion within the dynamic web of predisposing factors illustrated in the lower left quadrant of Figure 4. As investigators consider including any of these areas in experimental designs, future technological advances will continue to evolve, inform new approaches, advance discovery, and bolster the validity of OTS paradigms. These proposed areas are highlighted because all have been explored as complex systems and published using machine learning and other higher order analytics and modeling on high-dimensional data. Moreover, each topic represents a highly viable principle component in the web of predisposing factors for OTS (Figure 4).

### Brain Neural Networks and Machine Learning

Neuroscience is experiencing an ongoing rapid expansion of the scope and complexity of data collection/management/analysis with contemporary studies often collecting large volumes of data from networks that link multiple brain loci and cross multiple levels of organization and function (neurons, circuits, systems, and whole brain). The size and complexity of these data (i.e., in space and time) require sophisticated strategies for statistical inference and present important challenges for computing, data harmonization and sharing, and reproducibility.

Allen and colleagues (Allen et al., 2019) explored the nature of mouse brain activations in 2019, using implanted microelectrode arrays. Their research team recorded activations of >20,000 individual neurons, located at 34 rodent brain loci, during several hundred tasks. These procedures revealed a complex global system of neural networks and waves of activations across the brain (i.e., indicating the flow of information) that meet the criteria for a complex system (Table 1). Although generalizing the findings of rodent studies to humans must be done cautiously (Walsh, 1980), human brain researchers today acknowledge the existence of aggregates of networks that work across and within many levels of complexity and function. These experts envision that sensations, moods and behaviors are associated with hemispheric waves of neuronal activations that move in milliseconds (Armstrong and Kavouras, 2019), throughout a sprawling mass of 100 billion neurons which have at least 100 trillion connecting synapses (Bertolero and Bassett, 2019).

Investigators will need to take advantage of modern methods to analyze high-dimensional data, calculating probabilities and using pattern-searching algorithms that have been developed via advanced statistics and machine learning (Sejnowski et al., 2014). Notably, with advances in deep (machine) learning facilitated by artificial neural networks comprising ensembles of algorithms and models with brain-like logical structure and function, detailed characterization of the brain-wide neural activations/networks that are aligned with positive physiological adaptations to exercise training, as well as the maladaptations of nonfunctional overreaching and OTS, are likely on the near horizon.

### HPA Axis Dysfunction: Clues From Major Depression

The HPA and SAM axes are the primary regulators of human responses to virtually all life stresses (Figure 2). Both cortisol (HPA axis) and the catecholamines norepinephrine and epinephrine (SAM axis) are integrally involved in the responses to acute and chronic training stresses (see above section titled, Neuroendocrine Involvement in OTS). However, studies of neuroendocrine involvement in OTS are far from unanimous (Meeusen et al., 2013). This presently leads investigators to conclude that 1) measuring individual neuroendocrine variables cannot sufficiently capture the complexity of the body’s numerous simultaneous responses to any stressor (Schwartz, 2020), and 2) measuring a single or few variable(s) likely will not definitively identify athletes who are at risk or who exhibit OTS (Hooper et al., 2020).

The exact means by or degree to which neurotransmitters, neuromodulators and receptors contribute to or are affected by OTS remain unclear (Meeusen et al., 1999; Meeusen et al., 2013). However, numerous studies have focused on the roles of brain neurotransmitters and neuroendocrine responses in major depression (Schatzberg, 1998; Zunszain et al., 2011; Solomon et al., 2012; Nedic Erjavec et al., 2021). A 2017 study of major depressive disorder in a chronic-stress, depressive symptom murine model (Hervé et al., 2017) utilized integrated brain region-targeted transcriptomics, tissue-level global expression profiles, applied aspects of mice with symptoms, and pharmacological treatment or control conditions. Complex data analysis methods were employed to identify revealing signatures of complex system patterns distinguishing diseased, control, and effectively treated animals. Using similar approaches, this area of research offers useful insights into the nature of OTS because OTS and major depression exhibit similar signs and symptoms (Armstrong and VanHeest, 2002), suggesting a possible common etiology. For example, a considerable body of evidence shows that patients with major depression experience dysfunction of the HPA axis (Lechin et al., 1995; Maier and Watkins, 1998), as do athletes who exhibit OTS (Zautra and Reich, 1983; Barron et al., 1985; Fellmann et al., 1992; Urhausen et al., 1995; Keizer et al., 1998; Urhausen et al., 1998). Table 2 summarizes the similarities of signs, symptoms, and selected characteristics in OTS and major depression. Because these many similarities suggest a common etiology, we encourage investigators who design future OTS investigations to consider published research regarding the nature of major depression.

**TABLE 2:** Characteristics that have been associated with both the Overtraining Syndrome (OTS) and Major Depression (MD). Modified and updated from (Armstrong and VanHeest, 2002).

Characteristics	Supporting References	
	Overtraining Syndrome	Major Depression
A substantial variety of signs and symptoms are observed, with obvious inter-individual differences (OTS and MD). Signs and symptoms include general fatigue/malaise, insomnia, change of appetite, loss of motivation, reduced mental concentration, restlessness, loss of interest or pleasure in activities previously enjoyed, irritability (OTS and MD)	[Lehman et al. (1993), Froelich and Heil (1995), Smith (2000), Meeusen et al. (2013)]	Services. (1993); Epstein. (1997); van Eeden et al. (2020)
Clinical characteristics vary among individuals (OTS and MD) and exercise training regimens (OTS). Signs and symptoms are nonspecific and numerous (OTS and MD)	Verma et al. (1978); Fry et al. (1994); Morgan et al. (1988); Fry et al. (1991); Lehmann. (1992b); Lehman et al. (1993); Uusitalo et al. (1998)	Morgan et al. (1988); Eichner. (1995); Uusitalo et al. (1998)
Mental and physical performance are impaired (OTS and MD)	Keizer et al. (1998); Armstrong and VanHeest. (2002)	Wischnia. (1994a); Wischnia. (1994b); Armstrong and VanHeest. (2002)
Epigenetic changes are associated with this disorder (MD), exercise training (OTS), and with genes that regulate HPA axis function during stress (OTS)	(Alegría-Torres et al., 2011; Denham et al., 2014; Godoy, 2018; Saghiv and Sagiv, 2020; Silva, 2020)	Buschdorf and Meaney. (2015); Park et al. (2019)
Dysfunction of the HPA axis is involved, including altered glucocorticoid receptor sensitivity in the brain, reduced adrenal sensitivity to ACTH, and intensity/duration of neurotransmitter release (OTS and MD)^a^	Zautra and Reich. (1983; Lehmann. (1992b); Jakeman. (1994); Hooper. (1995); Urhausen et al. (1995); Foster. (1998); Fry et al. (1998); Keizer et al., 1998; Lehmann (1998); Urhausen et al. (1998); Meeusen et al. (2013)	Siever et al. (1986); Dey. (1994); Maier and Watkins. (1998); Schatzberg. (1998); Holsboer. (2000); Reichenberg et al. (2001); Leonard. (2006); McEwen. (2007); Zunszain et al. (2011); Solomon et al. (2012); Hughes et al. (2016); Camara and Brandao. (2020); Nedic Erjavec et al. (2021)
Chronic stress, acting via the HPA axis, is an important etiologic factor (OTS and MD)^a^	Wittert (1996); Armstrong and VanHeest (2002); Meeusen et al. (2010); Cadegiani and Kater (2020)	(Epstein, 1997; Leonard, 2006; Zunszain et al., 2011; Hughes et al., 2016)
Resolution requires weeks or months of rest (OTS and MD) or active rest (OTS)	Stone. (1991); Meeusen et al. (2013); Campbell and Turner (2018)	Services. (1993); Epstein. (1997); Ghosal et al. (2014); Jacobson. (2014); Mountjoy. (2014); Herman. (2016)
Immune system activation, cytokine release, and inflammation are involved (OTS and MD)	Morgan et al. (1988); Fry et al. (1991); Smith (2000); Meeusen et al. (2013)	Fent and Zbinden, (1987); Meijer et al. (1988); Irwin et al. (1990); Maes. (1993); Maes et al. (1997); Maier and Watkins. (1998); Reichenberg et al. (2001); Anisman. (2002); Leonard. (2006); Zunszain et al. (2011); Garate. (2013); Kelly et al. (2015); Nedic Erjavec et al. (2021)
Abnormalities of brain serotonergic function is an etiological factor (MD), and is theoretically related to fatigue (OTS)	Parry-Billings et al. (1990); Davis. (1995); Tanaka et al. (1997); Kreider et al. (1998b)	Meltzer. (1990); Azmitia and Whitaker-Azmitia. (1991); Jacobs and Azmitia. (1992); Dey. (1994); Maier and Watkins. (1998); Park et al. (2019)

a chronic stress may result in increased glucocorticoid secretion at rest, delayed reduction/termination of the stress response, facilitated or sensitized responses to novel stressors, or hyporesponsiveness in extreme cases (Siever et al., 1986; Herman, 2013; Jacobson, 2014). Abbreviations, HPA, hypothalamic-pituitary-adrenal axis; SAM, sympathetic-adrenal-medullary axis; ACTH, adrenocorticotrophic hormone

### The Intestinal Microbiota

The healthy human gut very likely contains more than 1,000 microorganism species (Hollister et al., 2014), each producing its own metabolites that interact with the intestinal ultrastructure, physiological functions, endocrine system, and the immune system (Armstrong et al., 2018). Although the totality of this widespread influence is unknown, primarily because the intestinal ecosystem remains incompletely characterized and its diversity poorly defined, the human intestinal microbiota (IM) is believed to exert measurable effects on metabolism (e.g., appetite control, energy balance (Huang et al., 2016), and diseases (e.g., obesity, diabetes, inflammatory bowel disease, neurological disorders, allergies, cancers, and cardiovascular diseases (Eckburg et al., 2005; Lozupone et al., 2012; Salonen and de Vos, 2014; Huang et al., 2016). In contrast, multiple positive health effects are imparted by the collective genome of the IM ecosystem (Wu et al., 2011; Ridaura et al., 2013), and specific microbe classes are considered especially important in human health and longevity (Wilmanski et al., 2021), owing to their effects on resilience to infection/inflammation, resistance to autoimmunity, and endocrine signaling; these classes include *Bacteroides, Bifidobacterium, Eubacterium, Faecalibacterium, Lactobacillus,* and *Roseburia* (Le Chatelier et al., 2013).

Two considerations regarding the composition/ratio of these IM bacterial classes are relevant to this review article. First, modifying one’s diet shifts the composition of the microbial ecosystem (Turnbaugh et al., 2007; Turnbaugh et al., 2009; David et al., 2014; Levy and Borenstein, 2014) and influences gut health (Slyepchenko et al., 2017). This effect was observed within 24 h, during a controlled feeding study of adults who consumed either a high-fat/low-fiber or a low-fat/high-fiber diet (Wu et al., 2011). Diet quality also influences immune function, systemic inflammation, and antioxidant capacity (for reviews of this topic see (Berk et al., 2013; Moylan et al., 2014). Second, individuals with a greater IM species diversity have a greater repertoire of microbial metabolic functions, as well as an IM population which is more functionally robust and theoretically enables and reinforces coping with homeostatic disruptions more effectively than individuals with less IM species diversity (Le Chatelier et al., 2013; Hollister et al., 2014). Thus, it is generally agreed that the characteristics of a healthy microbiota include community stability and increased species diversity (Campbell and Wisniewski, 2017; Armstrong et al., 2018).

Numerous studies have demonstrated that both acute and chronic diseases are associated with altered IM composition, reduced biodiversity, loss of commensal bacteria (i.e., accompanied by loss of beneficial metabolic activities such as nutrient bioavailability), and/or overgrowth of opportunistic pathogens (Ticinesi et al., 2019; Schmidt et al., 2018; Mosca et al., 2016; McKenzie et al., 2017; Marchesi et al., 2016; Levy et al., 2017; Kriss et al., 2018). Fortunately, a mild-to-moderate exercise training load serves as a potent intervention for the diversification of the gut IM independent of diet (Campbell and Wisniewski, 2017). Further, multiple human studies have assessed the effects of single bouts of exercise, or acute exercise periods spanning a few days (i.e., some of these employed metabolomic analyses, see Figure 5), on the composition of the IM (Clarke et al., 2014; Hold, 2014; Lambert et al., 2015; Mach and Fuster-Botella, 2017; O'Sullivan et al., 2015; Hsu et al., 2015; Clark and Mach, 2016; Bycura et al., 2021; Aya et al., 2021). Several studies have observed the acute effects of excessive exercise on the IM of mice (Allen et al., 2015; Yuan et al., 2018). Even the morbidity and mortality of exertional heatstroke are likely influenced by the IM, when strenuous exercise generates internal organ hyperthermia >40°C concurrent with endotoxemia (i.e., due to commensal Gram-negative bacteria or their byproducts translocating from the intestinal lumen into blood), hypotension, coma, and possible death (Armstrong et al., 2018). However, to our knowledge, longitudinal relationships between any aspect of the IM ecosystem and OTS or nonfunctional overreaching (Clark and Mach, 2016; Campbell and Wisniewski, 2017) have to-date not been published. Recent advances in complex wet-bench technology, biometrics, and data science have supported a robust evolution of the IM field, although the potential predisposing or consequent involvement with OTS remains unclear.

### Immune System Responses to Strenuous Training

Currently, the most compelling and relevant OTS theory related to the immune system (Smith, 2000) proposes that skeletal muscle, connective tissue, and joint microtrauma are compounded by inadequate rest and recovery, resulting in inflammation (Hackney and Lane, 2015). Proinflammatory cytokines (i.e., signaling molecules that mediate immune responses and inflammation; examples include interleukin-6 in certain scenarios, interleukin-1β, tumor necrosis factor-α), which are secreted by immune cells such as macrophages and T helper cells) act at multiple sites within the HPA axis, suggesting that cytokine release may underlie the neuroendocrine changes observed in OTS (MacKinnon, 2000). This theory states that, when microtrauma is chronic and excessive, the negative characteristics of OTS intensify and the athlete becomes physiologically and psychologically compromised (Meeusen et al., 2013; Hackney and Lane, 2015).

Because of insufficient and inconclusive scientific data, it is not known whether immune function is impaired in athletes who experience OTS. Although it is widely accepted that the immune system is sensitive to stress and that periods of intense training result in depressed immune cell function, these changes do not definitively distinguish athletes who successfully adapt to nonfunctional overreaching from those who maladapt and develop signs and symptoms of OTS (Meeusen et al., 2013). Similarly, Campbell and Turner (Campbell and Turner, 2018; Campbell and Turner, 2019) concluded that it is an oversimplification to conclude that acute exercise (e.g., a single bout of moderate-to-vigorous intensity endurance exercise) suppresses immune cell function and impairs overall immune competency.

We believe that immunological research to this date has been inconclusive because OTS is a complex clinical condition (Tables 1, 2) that 1) involves unique interactions of the host genome, multiple risk factors, and chronic stressors (Dhabhar and McEwen, 1997; Meeusen et al., 2013), 2) encompasses communication among cells of the immune system, brain, and skeletal muscle (Parry-Billings et al., 1990; Maier and Watkins, 1998), 3) produces highly individualized dysfunctional outcomes (MacKinnon, 2000; Bittencourt et al., 2016; Cadegiani and Kater, 2019b), and 4) has been studied using research designs which focus on one or a few variables. Thus, we propose that a complex systems approach (Figure 4) offers the best possibility of clarifying immune system roles and responses in athletes with OTS.

### Diet, Macronutrients, and Energy Availability

Few of the OTS studies in Supplementary Table S1 controlled athlete diets or measured habitual food and fluid intakes. To rectify this situation, as cited earlier, researchers evaluated 67 metabolic, dietary, hormonal, biochemical, anthropomorphic, and clinical variables in 51 men (aged 18–50 years; 14 athletes with OTS, 25 healthy athletes, and 12 healthy controls). Athletes with OTS were selected based on specific inclusion criteria, including verified underperformance. Statistically significant associations were identified between OTS and previously undiscovered dietary factors. Specifically, OTS was not a simple consequence of excessive training stress. However, it resulted from a combination of multiple risk factors and behaviors, including insufficient caloric, protein, and carbohydrate intake (Cadegiani and Kater, 2019b; Cadegiani and Kater, 2019c; Cadegiani and Sliva, 2020).

The 2013 joint consensus statement of the European College of Nutrition and the American College of Sports Medicine (Meeusen et al., 2013) noted that the fatigue and underperformance associated with nonfunctional overreaching (Figure 1) can be attributed, at least in part, to a low muscle glycogen concentration. This document also noted that 1) chronic energy deficiency and glycogen depletion amplify stress hormone (cortisol, norepinephrine/noradrenaline, epinephrine/adrenaline; Figure 2) and cytokine responses to exercise and may be determinants of emerging OTS, and 2) dietary carbohydrate supplementation may reverse the symptoms of nonfunctional overreaching (Meeusen et al., 2010). These two theoretical concepts were supported by research that reported reduced signs, symptoms, and performance decrements in runners who consumed a higher amount of carbohydrate for 7 days (5.4–8.5 g/kg body mass) during two iterations of strenuous training (Achten et al., 2004). Similarly, cyclists who undertook 8 days of intensive training twice, with a 2-weeks washout period between moderate and high carbohydrate diets, experienced smaller performance decrements, hormonal disturbances, altered moods, and number of days to recover from intensified training, when consuming a high carbohydrate diet (Halson et al., 1985).

Recently, an international team of physiologists and sport nutritionists published a narrative review paper that focused on the association between training overload and relative energy deficiency in sport (RED-S) (Stellingwerff, 2021). First introduced by the International Olympic Committee in 2014 (Mountjoy, 2014), RED-S is a complex syndrome caused by low energy availability that results in impaired physiological function and negative health/performance consequences. This team of experts summarized 21 training overload studies that measured energy intake and/or macronutrients (Stellingwerff, 2021). Most of these studies (86%) involved either decreased energy availability (i.e., intake minus expenditure), decreased carbohydrate availability, or a statistically significant difference between two cohorts. The authors 1) concluded that OTS and RED-S share many overlapping symptoms, 2) proposed that training-overload/OTS studies may be confounded by unrecognized inadequate energy intake (e.g., low dietary carbohydrates) coupled with high training loads, 3) cautioned that low energy availability leading to RED-S may be misdiagnosed as OTS, and 4) recommended that RED-S be evaluated and excluded before a diagnosis of OTS is given.

Nevertheless, not all athletes who undergo training overload experience decreased energy availability or reduced carbohydrate availability concurrent with RED-S symptoms (Lehmann, 1992a; Ramson, 2012). We interpret this observation to be the result of complex interactions of numerous predisposing factors (i.e., exercise mode, intensity, volume) in OTS (Supplementary Table S2, Figures 4, 5). Exploiting advancements in wet-bench high-throughput experimental approaches and computation and mathematical complex modeling and methods enables revisiting what is currently an ambiguous and at times contradictory body of understanding about diet, relative energy deficiency, and OTS. This will facilitate achieving clarity regarding the contributions of diet to OTS, the corresponding effect of OTS on subsequent eating behaviors, and the optimal nutrient and caloric intake to expedite recovery.

### Optimal Recovery From OTS

Just as determining the existence and severity of OTS is a complex process, so is developing an effective recovery plan for an affected athlete. Accordingly, novel, effective, and practical treatments for OTS are needed and thus are worthy of future study. Presently, the established and most successful treatment is rest with optimal nutrition, allowing the body to recover with the hope that performance competencies can be regained (Halson and Jeukendrup, 2004). However, a unique, need-based plan (i.e., especially one that individualizes the resumption of training and its frequency, duration, and intensity) is recommended for each athlete to optimize recovery and establish readiness to return to sport. Regular physical examinations by a physician, including laboratory tests, may identify upper respiratory tract infections or unresolved viral illnesses (Meeusen et al., 2013). A sport dietitian can assess an athlete’s energy and macronutrient deficiencies, and recommend changes of total caloric, carbohydrate, or protein intake (Lehmann et al., 1993; Lehmann et al., 1997; Cadegiani and Sliva, 2020; Stellingwerff, 2021). Even small within-day energy deficiencies (300–400 kcal/d), which usually do not impact immediate health outcomes, can become clinically significant when multiplied across months (Stellingwerff, 2021). If an athlete experiences (especially recurring and worsening) negative emotional or psychological changes during intense training overload, mental health can be monitored by a sports psychologist via communication, counseling, or mood questionnaires (Morgan et al., 1988). These requirements warrant the formation of a multi-disciplinary practitioner support team that holistically monitors health, performs scheduled multi-domain evaluations, diagnoses notable changes and concerns, and intervenes and treats early in response to signs and symptoms of emerging OTS (Meeusen et al., 2013; Stellingwerff, 2021). This integrated systems and multidisciplinary team approach is entirely consistent with the concept that OTS is a complex, multi-domain clinical condition (Supplementary Table S2, Figures 4, 5).

### A Go-Forward Guiding and Supporting Challenge

Each of the above domains (neural networks to recovery), accompanying rationales, and guiding approaches and principles for research, viably support and can measurably contribute to clarifying the nature and complex etiology of OTS. Moreover, researchers and clinicians are encouraged to shift from existing paradigms and limited experimental studies to confirm or refute specific reductionist hypotheses that foster single or only a few indicators to classify risk or confirm diagnosis of OTS. The challenging, though more rewarding, pursuit would be to embrace and utilize the advantages provided by the more revealing explanatory power of higher order and inclusive predictive models that more closely reflect the complex, dynamic, and integrated biological human systems, networks, and contributing factors underlying and that are fundamental to OTS.

## Conclusion

The definitive diagnosis and effective treatment of OTS remain elusive owing to its characteristic complexity, decidedly individualized phenotype, and inherent determinant interactions of numerous predisposing factors (e.g., the athlete’s genome, intense exercise training, and energy availability). As such, we recommend that future investigations employ methods appropriate to the analysis of complex, multi-domain, dynamic biological systems. Instead of looking for discrete or simply correlated individual determinants, a complex systems analysis enables exploring intrinsic (often non-linear) patterns of interaction among numerous potential predisposing factors. Once revealed, the probability of each pattern contributing to OTS (i.e., predict) is computed and accordingly ranked as features within the high-performing model(s). Consideration and selection of all high-probability features (adding great value to the OTS model) then allows a risk profile to be developed for an individual or a group of athletes. Instead of seeking cause-and-effect, this approach seeks the probability (i.e., risk) of OTS.

It is relevant to the present review paper that researchers in the specialty fields of Network Physiology and Complex Systems Science (Balagué et al., 2020) have proposed a new field of study (i.e., Network Physiology of Exercise) that focuses on the coordinated integration of physiological systems, subsystems, and network organization and topology to reveal how physiological adaptations and maladaptations emerge. These investigators also have proposed developing and applying new research methodologies, to quantify nonlinear dynamic connections among diverse systems, and establish basic principles of network organization and coordination among physiological systems. In concert with these approaches, the present review proposes that two methodological approaches will clarify the dynamics of physiological networks involved in OTS. First, assessing cellular functions during OTS across multiple levels of complexity (i.e., genomic, epigenetic, proteomic, and metabolomic characteristics). These trans-omic techniques will permit an analysis of thousands of contributing factors that lead to universal outcomes, which could not be detected by single biomarkers. Second, machine learning will pave the way for a paradigm shift away from hypothesis-driven experimental studies to a less intuitive predictive complex modeling schemes with robust practical application and flexible utility with new, unseen data.

With traditional past research methods applied to OTS, discerning the high-value predisposing risk factors remain unclear and conventional OTS prevention strategies and clinical management approaches have thus had limited consistent success. In contrast, the demonstrated advantages in examining more subtle and higher-order relationships in high-dimensional complex systems will mark a new advent of discovery and effective practical solutions for OTS.

## References

[B1] Achten J. Halson S. L. Moseley L. Rayson M. P. (2004). Higher Dietary Carbohydrate Content during Intensified Running Training Results in Better Maintenance of Performance and Mood State. J. Appl. Physiol. 96 (4), 1331–1340. 10.1152/japplphysiol.00973.2003 14660506

[B2] Aderem A. (2005). Systems Biology: its Practice and Challenges. Cell 121 (4), 511–513. 10.1016/j.cell.2005.04.020 15907465

[B3] Alegría-Torres J. A. Baccarelli A. Bollati V. (2011). Epigenetics and Lifestyle. Epigenomics 3 (3), 267–277. 10.2217/epi.11.22 22122337 PMC3752894

[B4] Allen J. M. Miller M. E. B. Pece B. D. Whitlock K. D. Nehra V. (2015). Voluntary and Forced Exercise Differentially Alters the Gut Microbiome in C57BL/6J Mice. J. Appl. Physiol. 118 (8), 1059–1066. 10.1152/japplphysiol.01077.2014 25678701

[B5] Allen W. E. Chen M. Z. Pichamoorthy N. Tien R. H. Pachitariu M. Luo L. (2019). Thirst Regulates Motivated Behavior through Modulation of Brainwide Neural Population Dynamics. Science 364 (6437), 253. 10.1126/science.aav3932 30948440 PMC6711472

[B6] Andrés-Rodríguez L. Borràs X. Feliu-Soler A. Pérez-Aranda A. Rozadilla-Sacanell A. Arranz B. (2019). Machine Learning to Understand the Immune-Inflammatory Pathways in Fibromyalgia. Int. J. Mol. Sci. 20 (17). 10.3390/ijms20174231 PMC6747258 31470635

[B7] Angione C. (2019). Human Systems Biology and Metabolic Modelling: A Review-From Disease Metabolism to Precision Medicine. Biomed. Res. Int. 2019, 8304260. 10.1155/2019/8304260 31281846 PMC6590590

[B8] Anisman H. (2002). Stress, Immunity, Cytokines and Depression. Acta Neuropsychiatr. 14 (6), 251–261. 10.1034/j.1601-5215.2002.140601.x 26984573

[B9] Armstrong L. E. Kavouras S. A. (2019). Thirst and Drinking Paradigms: Evolution from Single Factor Effects to Brainwide Dynamic Networks. Nutrients 11 (12). 10.3390/nu11122864 PMC6950074 31766680

[B10] Armstrong L. E. Lee E. C. Armstrong E. M. (2018). Interactions of Gut Microbiota, Endotoxemia, Immune Function, and Diet in Exertional Heatstroke. J. Sports Med. 2018, 5724575. 10.1155/2018/5724575 PMC5926483 29850597

[B11] Armstrong L. E. VanHeest J. L. (2002). The Unknown Mechanism of the Overtraining Syndrome. Sports Med. 32 (3), 185–209. 10.2165/00007256-200232030-00003 11839081

[B12] Aya V. Florez A. Perez L. (2021). Association between Physical Activity and Changes in Intestinal Microbiota Composition: A Systematic Review. PLoS One 16 (2), e0247039. 10.1371/journal.pone.0247039 33630874 PMC7906424

[B13] Azmitia E. C. Whitaker-Azmitia P. M. (1991). Awakening the Sleeping Giant: Anatomy and Plasticity of the Brain Serotonergic System. J. Clin. Psychiatry 52 (Suppl. l), 4–16. 1752858

[B14] Balagué N. Hristovski R. Almarcha M. Garcia-Retortillo S. Ivanov P. C. (2020). Network Physiology of Exercise: Vision and Perspectives. Front. Physiol. 11, 611550. 10.3389/fphys.2020.611550 33362584 PMC7759565

[B15] Bar-Yam Y. (2003). in Unifying Principles in Complex Systems, Technical Report. Editor Institute N. E. C. S. (Cambridge, MA), 1–33.

[B16] Barron J. L. Noakes T. D. Levy W. Smith C. Millar R. P. (1985). Hypothalamic Dysfunction in Overtrained Athletes*. J. Clin. Endocrinol. Metab. 60 (4), 803–806. 10.1210/jcem-60-4-803 2982908

[B17] Bashan A. Bartsch R. P. Kantelhardt J. W. Havlin S. Ivanov P. C. (2012). Network Physiology Reveals Relations between Network Topology and Physiological Function. Nat. Commun. 3, 702. 10.1038/ncomms1705 22426223 PMC3518900

[B18] Bergeron M. F. Landset S. Maugans T. A. Williams V. B. Collins C. L. Wasserman E. B. (2019). Machine Learning in Modeling High School Sport Concussion Symptom Resolve. Med. Sci. Sports Exerc. 51 (7), 1362–1371. 10.1249/mss.0000000000001903 30694980

[B19] Berk M. Williams L. J. Jacka F. N. O’Neil A. Pasco J. A. Moylan S. (2013). So Depression Is an Inflammatory Disease, but where Does the Inflammation Come from? BMC Med. 11, 200. 10.1186/1741-7015-11-200 24228900 PMC3846682

[B20] Bertolero M. Bassett D. (2019). How Matter Becomes Mind. Scientific American, 26–33. 10.1038/scientificamerican0719-26 39010450

[B21] Bittencourt N. F. N. Meeuwisse W. H. Mendonça L. D. Nettel-Aguirre A. Ocarino J. M. Fonseca S. T. (2016). Complex Systems Approach for Sports Injuries: Moving from Risk Factor Identification to Injury Pattern Recognition-Narrative Review and New Concept. Br. J. Sports Med. 50 (21), 1309–1314. 10.1136/bjsports-2015-095850 27445362

[B22] Breuner C. W. Delehanty B. Boonstra R. (2012). Evaluating Stress in Natural Populations of Vertebrates: Total CORT Is Not Good Enough. Funct. Ecol. 27, 24–36. 10.1111/1365-2435.12016

[B23] Brustmann M. Hoske H. (1928). Normale und pathologische physiologie der leibesȕebungen. Müenchener Medizinische Wochenschrift 251, 1834.

[B24] Budgett R. (1998). Fatigue and Underperformance in Athletes: the Overtraining Syndrome. Br. J. Sports Med. 32 (2), 107–110. 10.1136/bjsm.32.2.107 9631215 PMC1756078

[B25] Buldyrev S. V. Parshani R. Paul G. (2010). Catastrophic cascade of Failures in Interdependent Networks. Nature 464 (7291), 1025–1028. 10.1038/nature08932 20393559

[B26] Buschdorf J. P. Meaney M. J. (2015). Epigenetics/Programming in the HPA Axis. Compr. Physiol. 6 (1), 87–110. 10.1002/cphy.c140027 26756628

[B27] Buyse L. (2019). Improving the Diagnosis of Nonfunctional Overreaching and Overtraining Syndrome. Med. Sci. Sports Exerc. 51 (12), 2524–2530. 10.1249/mss.0000000000002084 31274684

[B28] Bycura D. Santos A. C. Shiffer A. Kyman S. Winfree K. Sutliffe J. (2021). Impact of Different Exercise Modalities on the Human Gut Microbiome. Sports (Basel) 9, 14. 10.3390/sports9020014 33494210 PMC7909775

[B29] Cadegiani F. A. Kater C. E. (2019b). Basal Hormones and Biochemical Markers as Predictors of Overtraining Syndrome in Male Athletes: The EROS-BASAL Study. J. Athl Train. 54 (8), 906–914. 10.4085/1062-6050-148-18 31386577 PMC6756603

[B30] Cadegiani F. A. Kater C. E. (2018a). Body Composition, Metabolism, Sleep, Psychological and Eating Patterns of Overtraining Syndrome: Results of the EROS Study (EROS-PROFILE). J. Sports Sci. 36 (16), 1902–1910. 10.1080/02640414.2018.1424498 29313445

[B31] Cadegiani F. A. Kater C. E. (2020). Eating, Sleep, and Social Patterns as Independent Predictors of Clinical, Metabolic, and Biochemical Behaviors Among Elite Male Athletes: The EROS-PREDICTORS Study. Front. Endocrinol. 11, 414. 10.3389/fendo.2020.00414 PMC7332731 32670198

[B32] Cadegiani F. A. Kater C. E. (2017). Hormonal Aspects of Overtraining Syndrome: a Systematic Review. BMC Sports Sci. Med. Rehabil. 9, 14. 10.1186/s13102-017-0079-8 28785411 PMC5541747

[B33] Cadegiani F. A. Kater C. E. (2018b). Hormonal Response to a Non-exercise Stress Test in Athletes with Overtraining Syndrome: Results from the Endocrine and Metabolic Responses on Overtraining Syndrome (EROS) - EROS-STRESS. J. Sci. Med. Sport 21 (7), 648–653. 10.1016/j.jsams.2017.10.033 29157780

[B34] Cadegiani F. A. Kater C. E. (2019c). Novel Causes and Consequences of Overtraining Syndrome: the EROS-DISRUPTORS Study. BMC Sports Sci. Med. Rehabil. 11, 21. 10.1186/s13102-019-0132-x 31548891 PMC6751688

[B35] Cadegiani F. A. Kater C. E. (2019a). Novel Insights of Overtraining Syndrome Discovered from the EROS Study. BMJ Open Sport Exerc. Med. 5 (1), e000542. 10.1136/bmjsem-2019-000542 PMC6590962 31297238

[B36] Cadegiani F. A. Sliva P. H. F. D. (2020). Diagnosis of Overtraining Syndrome: Results of the Endocrine and Metabolic Responses on Overtraining Syndrome Study: EROS-DIAGNOSIS. J. Sports Med. 2020, 3937819. 10.1155/2020/3937819 PMC7193300 32373644

[B37] Camara A. B. Brandao I. A. (2020). Neural Receptors Associated with Depression: A Systematic Review of the Past 10 Years. CNS Neurol. Disord. Drug Targets 19 (6), 417–436. 10.2174/1871527319666200715102430 32669081

[B38] Campbell J. P. Turner J. E. (2018). Debunking the Myth of Exercise-Induced Immune Suppression: Redefining the Impact of Exercise on Immunological Health across the Lifespan. Front. Immunol. 9, 648. 10.3389/fimmu.2018.00648 29713319 PMC5911985

[B39] Campbell J. P. Turner J. E. (2019). There Is Limited Existing Evidence to Support the Common assumption that Strenuous Endurance Exercise Bouts Impair Immune Competency. Expert Rev. Clin. Immunol. 15 (2), 105–109. 10.1080/1744666x.2019.1548933 30430884

[B40] Campbell S. C. Wisniewski P. J. (2017). Exercise Is a Novel Promoter of Intestinal Health and Microbial Diversity. Exerc. Sport Sci. Rev. 45 (1), 41–47. 10.1249/jes.0000000000000096 27782912

[B41] Carter J. G. Potter A. W. Brooks K. A. (2014). Overtraining Syndrome: Causes, Consequences, and Methods for Prevention. J. Sport Human Perf. 2 (1), 1–14. 10.12922/jshp.0031.2014

[B42] Civelek M. Lusis A. J. (2014). Systems Genetics Approaches to Understand Complex Traits. Nat. Rev. Genet. 15 (1), 34–48. 10.1038/nrg3575 24296534 PMC3934510

[B43] Clark A. Mach N. (2016). Exercise-induced Stress Behavior, Gut-Microbiota-Brain axis and Diet: a Systematic Review for Athletes *.* J. Int. Soc. Sports Nutr. 13, 43. 10.1186/s12970-016-0155-6 27924137 PMC5121944

[B44] Clarke S. F. Murphy E. F. O'Sullivan O. Lucey A. J. Humphreys M. Hogan A. (2014). Exercise and Associated Dietary Extremes Impact on Gut Microbial Diversity. Gut 63 (12), 1913–1920. 10.1136/gutjnl-2013-306541 25021423

[B45] Coffey D. S. (1998). Self-organization, Complexity and Chaos: the New Biology for Medicine. Nat. Med. 4 (8), 882–885. 10.1038/nm0898-882 9701230

[B46] Costill D. L. (1988). Effects of Repeated Days of Intensified Training on Muscle Glycogen and Swimming Performance. Med. Sci. Sports Exerc. 20 (3), 249–254. 10.1249/00005768-198806000-00006 3386503

[B47] Counsilman J. (1955). Fatigue and Staleness. Athletic J. 15, 16–20.

[B48] David L. A. Maurice C. F. Carmody R. N. Gootenberg D. B. Button J. E. Wolfe B. E. (2014). Diet Rapidly and Reproducibly Alters the Human Gut Microbiome. Nature 505 (7484), 559–563. 10.1038/nature12820 24336217 PMC3957428

[B49] Davis J. M. (1995). Carbohydrates, Branched-Chain Amino Acids, and Endurance: the central Fatigue Hypothesis. Int. J. Sport Nutr. 5 (Suppl. l), S29–S38. 10.1123/ijsn.5.s1.s29 7550256

[B50] Denham J. Marques F. Z. O’Berien B. J. Chaarcar F. J. (2014). Exercise: Putting Action into Our Epigenome. Sports Med. 44 (2), 189–209. 10.1007/s40279-013-0114-1 24163284

[B51] Deussing J. M. Chen A. (2018). The Corticotropin-Releasing Factor Family: Physiology of the Stress Response. Physiol. Rev. 98 (4), 2225–2286. 10.1152/physrev.00042.2017 30109816

[B52] Dey S. (1994). Physical Exercise as a Novel Antidepressant Agent: Possible Role of Serotonin Receptor Subtypes. Physiol. Behav. 55 (2), 323–329. 10.1016/0031-9384(94)90141-4 8153173

[B53] Dhabhar F. S. McEwen B. S. (1997). Acute Stress Enhances while Chronic Stress Suppresses Cell-Mediated Immunity *In Vivo*: a Potential Role for Leukocyte Trafficking. Brain Behav. Immun. 11 (4), 286–306. 10.1006/brbi.1997.0508 9512816

[B54] Dickens M. J. Romero L. M. (2013). A Consensus Endocrine Profile for Chronically Stressed Wild Animals Does Not Exist. Gen. Comp. Endocrinol. 191, 177–189. 10.1016/j.ygcen.2013.06.014 23816765

[B55] Eckburg P. B. Bik E. M. Bernstein C. N. Purdom E. Dethlefsen L. Sargent M. (2005). Diversity of the Human Intestinal Microbial flora. Science 308 (5728), 1635–1638. 10.1126/science.1110591 15831718 PMC1395357

[B56] Eichner E. R. (1995). Overtraining: Consequences and Prevention. J. Sports Sci. 13 (Suppl. 1), S41–S48. 10.1080/02640419508732276 8897319

[B57] Epstein Y. (1997). Heat Intolerance Induced by Antidepressants. Ann. N. Y Acad. Sci. 813, 553–558. 10.1111/j.1749-6632.1997.tb51746.x 9100934

[B58] Fellmann N. Bedu M. Boudet G. Mage M. Sagnol M. Pequignot J.-M. (1992). Inter-relationships between Pituitary-Adrenal Hormones and Catecholamines during a 6-day Nordic Ski Race. Europ. J. Appl. Physiol. 64 (3), 258–265. 10.1007/bf00626289 1314173

[B59] Fent K. Zbinden G. (1987). Toxicity of Interferon and Interleukin. Trends Pharmacol. Sci. 8 (3), 100–105. 10.1016/0165-6147(87)90083-6

[B60] Fleck S. J. Kraemer W. J. (1982). The Overtraining Syndrome. Strength Conditioning J. 4 (4). 10.1519/0199-610x(1982)004<0050:tos>2.3.co;2

[B61] Foster C. (1998). Monitoring Training in Athletes with Reference to Overtraining Syndrome. Med. Sci. Sports Exerc. 30 (7), 1164–1168. 10.1097/00005768-199807000-00023 9662690

[B62] Freckleton G. Pizzari T. (2013). Risk Factors for Hamstring Muscle Strain Injury in Sport: a Systematic Review and Meta-Analysis. Br. J. Sports Med. 47 (6), 351–358. 10.1136/bjsports-2011-090664 22763118

[B63] Froelich J. (1995). Overtraining Syndrome, in Psychology of Sport Injury, Editor Heil J., Human Kinetics: Champaign, IL. p. 59–70.

[B64] Fry A. C. Kraemer W. J. Van Borselen F. Lynch J. M. Triplett N. T. Koziris L. P. (1994). Catecholamine Responses to Short-Term High-Intensity Resistance Exercise Overtraining. J. Appl. Physiol. (1985) 77 (2), 941–946. 10.1152/jappl.1994.77.2.941 8002551

[B65] Fry A. C. (2006). beta2-Adrenergic Receptor Downregulation and Performance Decrements during High-Intensity Resistance Exercise Overtraining. J. Appl. Physiol. 101 (6), 1664–1672. 10.1152/japplphysiol.01599.2005 16888042

[B66] Fry A. C. Kraemer W. J. (1997). Resistance Exercise Overtraining and Overreaching. Sports Med. 23 (2), 106–129. 10.2165/00007256-199723020-00004 9068095

[B67] Fry A. C. (1998). “The Role of Training Intensity in Resistance Exercise Overtraining and Overreaching,” in Overtraining in Sport. Human Kinetics. Editors Kreider R. B. Fry A. C. O'Toole M. L. (Champaign, IL, US), 403.

[B68] Fry A. Steinacker J. Meeusen R. (2005). “Endocrinology of Overtraining,” in The Endocrine System in Sports and Exercise. Editors Kraemer E. W. J. Robergs R. (Malden, MA: Blackwell Scientific), 578–599.

[B69] Fry R. W. Morton A. R. Keast D. (1991). Overtraining in Athletes. Sports Med. 12 (1), 32–65. 10.2165/00007256-199112010-00004 1925188

[B70] Garate I. (2013). Stress-induced Neuroinflammation: Role of the Toll-like Receptor-4 Pathway. Biol. Psychiatry 73 (1), 32–43. 10.1016/j.biopsych.2012.07.005 22906518

[B71] Ghosal S. Oscaka J. B. MDoglas C. Myres B. (2014). Glucocorticoid Receptors in the Nucleus of the Solitary Tract (NTS) Decrease Endocrine and Behavioral Stress Responses. Psychoneuroendocrinology 45, 142–153. 10.1016/j.psyneuen.2014.03.018 24845185 PMC4076411

[B72] Godoy L. D. (2018). A Comprehensive Overview on Stress Neurobiology: Basic Concepts and Clinical Implications. Front. Behav. Neurosci. 12, 127. 10.3389/fnbeh.2018.00127 30034327 PMC6043787

[B73] Goldstein D. S. Kopin I. J. (2008). Adrenomedullary, Adrenocortical, and Sympathoneural Responses to Stressors: a Meta-Analysis. Endocr. Regul. 42 (4), 111–119. 18999898 PMC5522726

[B74] Hackney A. C. Lane A. R. (2015). Exercise and the Regulation of Endocrine Hormones. Prog. Mol. Biol. Transl Sci. 135, 293–311. 10.1016/bs.pmbts.2015.07.001 26477919

[B75] Häkkinen K. Keskinen K. L. Alén M. Komi P. V. Kauhanen H. (1989). Serum Hormone Concentrations during Prolonged Training in Elite Endurance-Trained and Strength-Trained Athletes. Europ. J. Appl. Physiol. 59 (3), 233–238. 10.1007/bf02386193 2583168

[B76] Halson S. L. Jeukendrup A. E. (2004). Does Overtraining Exist? Sports Med. 34 (14), 967–981. 10.2165/00007256-200434140-00003 15571428

[B77] Halson S. L. Lancaster J. I. Acchten J. (1985). Effects of Carbohydrate Supplementation on Performance and Carbohydrate Oxidation after Intensified Cycling Training. J. Appl. Physiol. 97 (4), 1245–1253. 10.1152/japplphysiol.01368.2003 15155717

[B78] Hastie T. Friedman J. Tibshirani R. (2001). “Overview of Supervised Learning,” in The Elements of Statistical Learning (New York: Springer), 9–40. 10.1007/978-0-387-21606-5_2

[B79] Herman J. P. (2013). Neural Control of Chronic Stress Adaptation. Front. Behav. Neurosci. 7, 61. 10.3389/fnbeh.2013.00061 23964212 PMC3737713

[B80] Herman J. P. (2016). Regulation of the Hypothalamic-Pituitary-Adrenocortical Stress Response. Compr. Physiol. 6 (2), 603–621. 10.1002/cphy.c150015 27065163 PMC4867107

[B81] Hervé M. Bergon A. Le Guisquet A.-M. Leman S. Consoloni J.-L. Fernandez-Nunez N. (2017). Translational Identification of Transcriptional Signatures of Major Depression and Antidepressant Response. Front. Mol. Neurosci. 10, 248. 10.3389/fnmol.2017.00248 28848385 PMC5550836

[B82] Higgins J. (2003). Nonlinear Systems in Medicine. Yale J. Biol. Med. 75, 247–260. PMC2588816 14580107

[B83] Hold G. L. (2014). The Gut Microbiota, Dietary Extremes and Exercise. Gut 63 (12), 1838–1839. 10.1136/gutjnl-2014-307305 25021422

[B84] Holland J. (1995). Hidden Order: How Adaptation Builds Complexity from Chaos. Reading, MA: Perseus Books.

[B85] Hollister E. B. Gao C. Versalovic J. (2014). Compositional and Functional Features of the Gastrointestinal Microbiome and Their Effects on Human Health. Gastroenterology 146 (6), 1449–1458. 10.1053/j.gastro.2014.01.052 24486050 PMC4181834

[B86] Holsboer F. (2000). The Corticosteroid Receptor Hypothesis of Depression. Neuropsychopharmacology 23 (5), 477–501. 10.1016/s0893-133x(00)00159-7 11027914

[B87] Hooper D. Snyder A. Hackney A. (2020). “The Endocrine System in Overtraining,” in Endocrinology of Physical Activity and Sport. Editor Constantini A. H. a. N. (Cham, Switzerland: Springer Nature), 494–506. 10.1007/978-3-030-33376-8_27

[B88] Hooper S. L. Mackinnon L. T. Gordon R. D. Bachmann A. W. (1993). Hormonal Responses of Elite Swimmers to Overtraining. Med. Sci. Sports Exerc. 25 (6), 741–747. 10.1249/00005768-199306000-00015 8321113

[B89] Hooper S. L. (1995). Markers for Monitoring Overtraining and Recovery. Med. Sci. Sports Exerc. 27 (1), 106–112. 10.1249/00005768-199501000-00019 7898325

[B90] Hristovski R. (2010). Constraints-controlled Metastable Dynamics of Exercise-Induced Psychobiological Adaptation. Medicina (Kaunas) 46 (7), 447–453. 10.3390/medicina46070064 20966616

[B91] Hsu Y. J. Chiu C. C. Li Y. P. Huang W. C. Huang Y. T. Huang C. C. (2015). Effect of Intestinal Microbiota on Exercise Performance in Mice. J. Strength Cond Res. 29 (2), 552–558. 10.1519/jsc.0000000000000644 25144131

[B92] Huang H. Krishnan H. B. Pham Q. Yu L. L. Wang T. T. Y. (2016). Soy and Gut Microbiota: Interaction and Implication for Human Health. J. Agric. Food Chem. 64 (46), 8695–8709. 10.1021/acs.jafc.6b03725 27798832

[B93] Hughes M. M. Connor T. J. Harkin A. (2016). Stress-Related Immune Markers in Depression: Implications for Treatment. Int. J. Neuropsychopharmacol. 19 (6), pyw001. 10.1093/ijnp/pyw001 26775294 PMC4926799

[B94] Irwin M. Schuman G. Bustillo J. Turner J. A. Zhiang R. Zhi D. (1990). Reduction of Immune Function in Life Stress and Depression. Biol. Psychiatry 27 (1), 22–30. 10.1016/0006-3223(90)90016-u 2297549

[B95] Ivanov P. C. Bartsch R. P. (2014). “Network Physiology: Mapping Interactions between Networks of Physiologic Networks,” in Networks of Networks: The Last Frontier of Complexity. Editor Scala G. D. A. a. A. (Cham, Switzerland: Springer International), 203–222. 10.1007/978-3-319-03518-5_10

[B96] Jacobs B. L. Azmitia E. C. (1992). Structure and Function of the Brain Serotonin System. Physiol. Rev. 72 (1), 165–229. 10.1152/physrev.1992.72.1.165 1731370

[B97] Jacobson L. (2014). Hypothalamic-pituitary-adrenocortical axis: Neuropsychiatric Aspects. Compr. Physiol. 4 (2), 715–738. 10.1002/cphy.c130036 24715565

[B98] Jakeman P. M. (1994). Evidence for Downregulation of Hypothalamic 5-hydroxytryptamine Receptor Function in Endurance-Trained Athletes. Exp. Physiol. 79 (3), 461–464. 10.1113/expphysiol.1994.sp003780 8074858

[B99] Jeukendrup A. E. (1992). Physiological Changes in Male Competitive Cyclists after Two Weeks of Intensified Training. Int. J. Sports Med. 13 (7), 534–541. 10.1055/s-2007-1021312 1459749

[B100] Keizer H. (1998). Neuroendocrine Aspects of Overtraining. Overtraining in Sport. Human Kinetics. Editors Kreider R. B. Fry A. C. O'Toole M. L. Champaign, IL, US, 403.

[B101] Kelly J. R. Kennedy P. J. Cryan J. F. Ginan T. J. (2015). Breaking Down the Barriers: the Gut Microbiome, Intestinal Permeability and Stress-Related Psychiatric Disorders. Front Cel Neurosci 9, 392. 10.3389/fncel.2015.00392 PMC4604320 26528128

[B102] Kereszty A. (1947). A. Letörésröl. Sportorvos 1, 16.

[B103] Kereszty A. (1971). Overtraining, in Encyclopedia of Sport Sciences and Medicine. Editor Larson L., McMillan: New York. p. 218–222.

[B104] Knoll W. Arnold A. (1933). Normale und pathologische physiologie der leibesȕebungen. Editor Barth J. A. Munich.

[B105] Koutedakis Y. Budgett R. Faulmann L. (1990). Rest in Underperforming Elite Competitors. Br. J. Sports Med. 24 (4), 248–252. 10.1136/bjsm.24.4.248 2097024 PMC1478905

[B106] Kraemer W. Nindl B. (1998). “Factors Involved with Overtraining for Strength and Power, in Overtraining in Sport, Editor Kreider A. C. F. R. B. O’Toole M. L., Human Kinetics: Champaign, IL. p. 69–86.

[B107] Kraemer W. J. Fleck S. J. Callister R. Shealy M. Dudley G. A. Maresh C. M. (1989). Training Responses of Plasma Beta-Endorphin, Adrenocorticotropin, and Cortisol. Med. Sci. Sports Exerc. 21 (2), 146–153. 10.1249/00005768-198904000-00006 2540392

[B108] Kraemer W. J. Ratamess N. A. (2005). Hormonal Responses and Adaptations to Resistance Exercise and Training. Sports Med. 35 (4), 339–361. 10.2165/00007256-200535040-00004 15831061

[B109] Kreider R. Fry A. O'Toole M. (1998a). Overtraining in Sport: Terms, Definitions, and Prevalence, in Overtraining in Sport, Editor Kreider A. C. F. R. O’Toole M., Human Kinetics: Champaign, IL.

[B110] Kreider R. B. Fry A. C. O'Toole M. L. (1998b). “Overtraining in Sport,” in Overtraining in Sport. Human Kinetics. Editors Kreider R. B. Fry A. C. O'Toole M. L. (Champaign, IL, US, 403. 10.1097/00005768-199805001-01277

[B111] Kriss M. Hazleton K. Z. Nusbacher N. M. Martin C. G. Lozupone C. A. (2018). Low Diversity Gut Microbiota Dysbiosis: Drivers, Functional Implications and Recovery. Curr. Opin. Microbiol. 44, 34–40. 10.1016/j.mib.2018.07.003 30036705 PMC6435260

[B112] Kuipers H. Keizer H. A. (1988). Overtraining in Elite Athletes. Sports Med. 6 (2), 79–92. 10.2165/00007256-198806020-00003 3062735

[B113] Lachuer J. Delton I. Buda M. Tappaz M. (1994). The Habituation of Brainstem Catecholaminergic Groups to Chronic Daily Restraint Stress Is Stress Specific like that of the Hypothalamo-Pituitary-Adrenal axis. Brain Res. 638 (1-2), 196–202. 10.1016/0006-8993(94)90650-5 8199859

[B114] Lambert J. E. Myslicki J. P. Bomhof M. R. Belke D. D. Shearer J. Reimer R. A. (2015). Exercise Training Modifies Gut Microbiota in normal and Diabetic Mice. Appl. Physiol. Nutr. Metab. 40 (7), 749–752. 10.1139/apnm-2014-0452 25962839

[B115] Le Chatelier E. Nielsen T. Qin J. Prifti E. Hildebrand F. (2013). Richness of Human Gut Microbiome Correlates with Metabolic Markers. Nature 500 (7464), 541–546. 10.1038/nature12506 23985870

[B116] Lechin F. van der Dijs B. Orozco B. Lechin M. E. Báez S. Lechin A. E. (1995). Plasma Neurotransmitters, Blood Pressure, and Heart Rate during Supine-Resting, Orthostasis, and Moderate Exercise Conditions in Major Depressed Patients. Biol. Psychiatry 38 (3), 166–173. 10.1016/0006-3223(94)00258-5 7578659

[B117] Lehman M. Foster C. Keul J. (1993). Overtraining in Endurance Athletes: a Brief Review. Med. Sci. Sports Exerc. 25 (7), 854–862. 10.1249/00005768-199307000-00015 8350709

[B118] Lehmann M. (1998). Autonomic Imbalance Hypothesis and Overtraining Syndrome. Med. Sci. Sports Exerc. 30 (7), 1140–1145. 10.1097/00005768-199807000-00019 9662686

[B119] Lehmann M. Dickuth H. H. (1991). Training-overtraining. A Prospective, Experimental Study with Experienced Middle- and Long-Distance Runners. Int. J. Sports Med. 12 (5), 444–452. 10.1055/s-2007-1024711 1752709

[B120] Lehmann M. J. Lormes W. Opitz-Gress A. Steinacker J. M. Netzer N. Foster C. (1997). Training and Overtraining: an Overview and Experimental Results in Endurance Sports. J. Sports Med. Phys. Fitness 37 (1), 7–17. 9190120

[B121] Lehmann M. Knizia K. Gastmann U. Petersen K. G. Khalaf A. N. Bauer S. (1993). Influence of 6-week, 6 Days Per Week, Training on Pituitary Function in Recreational Athletes. Br. J. Sports Med. 27 (3), 186–192. 10.1136/bjsm.27.3.186 8242277 PMC1332185

[B122] Lehmann M. (1998). Physiological Responses to Short- and Long-Term Overtraining in Endurance Athletes, in Overtraining in Sport, Editors Kreider A. C. F. R. B. O’Toole M. L., Human Kinetics: Champaign, IL. p. 19–46.

[B123] Lehmann M. (1999).Selected Parameters and Mechanisms of Peripheral and central Fatigue and Regeneration in Overtrained Athletes, in Overload, Performance Incompetence, and Regeneration in Sport, Editors Lehmann C. F. M. Gastmann U. Keizer H. Steinacker J. M., Springer: Boston. p. 7–25.

[B124] Lehmann M. (1992). Training-overtraining: Influence of a Defined Increase in Training Volume vs Training Intensity on Performance, Catecholamines and Some Metabolic Parameters in Experienced Middle- and Long-Distance Runners. Eur. J. Appl. Physiol. Occup. Physiol. 64 (2), 169–177. 10.1007/bf00717956 1555564

[B125] Lehmann M. (1992). Training-overtraining: Performance, and Hormone Levels, after a Defined Increase in Training Volume versus Intensity in Experienced Middle- and Long-Distance Runners. Br. J. Sports Med. 26 (4), 233–242. 10.1136/bjsm.26.4.233 1490214 PMC1479002

[B126] Leonard B. E. (2006). HPA and Immune Axes in Stress: Involvement of the Serotonergic System. Neuroimmunomodulation 13 (5-6), 268–276. 10.1159/000104854 17709948

[B127] Levy M. Kolodziejczyk A. A. Thaiss C. A. Elinav E. (2017). Dysbiosis and the Immune System. Nat. Rev. Immunol. 17 (4), 219–232. 10.1038/nri.2017.7 28260787

[B128] Levy R. Borenstein E. (2014). Metagenomic Systems Biology and Metabolic Modeling of the Human Microbiome. Gut Microbes 5 (2), 265–270. 10.4161/gmic.28261 24637600 PMC4063856

[B129] Lozupone C. A. Stombaugh J. I. Gordon J. I. Jansson J. K. Knight R. (2012). Diversity, Stability and Resilience of the Human Gut Microbiota. Nature 489 (7415), 220–230. 10.1038/nature11550 22972295 PMC3577372

[B130] MacDougall-Shackleton S. A. Bonier F. Romero L. M. Moore I. T. (2019). Glucocorticoids and "Stress" Are Not Synonymous. Integr. Org. Biol. 1 (1), obz017. 10.1093/iob/obz017 33791532 PMC7671118

[B131] Mach N. Fuster-Botella D. (2017). Endurance Exercise and Gut Microbiota: A Review. J. Sport Health Sci. 6 (2), 179–197. 10.1016/j.jshs.2016.05.001 30356594 PMC6188999

[B132] MacKinnon L. T. (2000). Overtraining Effects on Immunity and Performance in Athletes. Immunol. Cel Biol 78 (5), 502–509. 10.1111/j.1440-1711.2000.t01-7-.x 11050533

[B133] Maes M. (1993). Psychomotor Retardation, Anorexia, Weight Loss, Sleep Disturbances, and Loss of Energy: Psychopathological Correlates of Hyperhaptoglobinemia during Major Depression. Psychiatry Res. 47 (3), 229–241. 10.1016/0165-1781(93)90081-q 8372161

[B134] Maes M. Verkerk R. Van Hunsel F. Neels H. (1997). Serotonin-immune Interactions in Major Depression: Lower Serum Tryptophan as a Marker of an Immune-Inflammatory Response. Eur. Arch. Psychiatry Clin. Neurosci. 247 (3), 154–161. 10.1007/bf03033069 9224908

[B135] Maier S. F. Watkins L. R. (1998). Cytokines for Psychologists: Implications of Bidirectional Immune-To-Brain Communication for Understanding Behavior, Mood, and Cognition. Psychol. Rev. 105 (1), 83–107. 10.1037/0033-295x.105.1.83 9450372

[B136] Main L. Grove J. R. (2009). A Multi-Component Assessment Model for Monitoring Training Distress Among Athletes. Eur. J. Sport Sci. 9 (4), 195–202. 10.1080/17461390902818260

[B137] Marchesi J. R. Adams D. H. Fava F. Hermes G. D. A. Hirschfield G. M. Hold G. (2016). The Gut Microbiota and Host Health: a New Clinical Frontier. Gut 65 (2), 330–339. 10.1136/gutjnl-2015-309990 26338727 PMC4752653

[B138] McEwen B. S. (2007). Physiology and Neurobiology of Stress and Adaptation: central Role of the Brain. Physiol. Rev. 87 (3), 873–904. 10.1152/physrev.00041.2006 17615391

[B139] McKenzie C. Tan J. Macia L. Mackay C. R. (2017). The Nutrition-Gut Microbiome-Physiology axis and Allergic Diseases. Immunol. Rev. 278 (1), 277–295. 10.1111/imr.12556 28658542

[B140] Meeusen R. (1999). Overtraining And the central Nervous System: The Missing Link? Overload, Performance Incompetence, and Regeneration in Sport. Editors Lehmann C. F. Gastmann U. Keizer H. Steinacker J. M. (Springer).

[B141] Meeusen R. Duclos M. Foster C. Fry A. Gleeson M. Nieman D. (2013). Prevention, Diagnosis, and Treatment of the Overtraining Syndrome: Joint Consensus Statement of the European College of Sport Science and the American College of Sports Medicine. Med. Sci. Sports Exerc. 45 (1), 186–205. 10.1249/MSS.0b013e318279a10a 23247672

[B142] Meeusen R. (2007). Brain Neurotransmitters in Fatigue and Overtraining. Appl. Physiol. Nutr. Metab. 32 (5), 857–864. 10.1139/h07-080 18059610

[B143] Meeusen R. (2004). Hormonal Responses in Athletes: the Use of a Two Bout Exercise Protocol to Detect Subtle Differences in (Over)training Status. Eur. J. Appl. Physiol. 91 (2-3), 140–146. 10.1007/s00421-003-0940-1 14523562

[B144] Meeusen R. Nederhof E. Buyse L. Roelands B. de Schutter G. Piacentini M. F. (2010). Diagnosing Overtraining in Athletes Using the Two-Bout Exercise Protocol. Br. J. Sports Med. 44 (9), 642–648. 10.1136/bjsm.2008.049981 18703548

[B145] Meeuwisse W. H. Tyreman H. Hagel B. Emery C. (2007). A Dynamic Model of Etiology in Sport Injury: the Recursive Nature of Risk and Causation. Clin. J. Sport Med. 17 (3), 215–219. 10.1097/jsm.0b013e3180592a48 17513916

[B146] Meijer A. Zakay-Rones Z. Morag A. (1988). Post-influenzal Psychiatric Disorder in Adolescents. Acta Psychiatr. Scand. 78 (2), 176–181. 10.1111/j.1600-0447.1988.tb06319.x 3223316

[B147] Mellerowicz H. Barron D. (1971) Overtraining, in Encyclopedia of Sport Sciences and Medicine. Editors Larson A., McMillan: New York. p. 1310–1312.

[B148] Meltzer H. Y. (1990). Role of Serotonin in Depression. Ann. N. Y Acad. Sci. 600, 486–499. 10.1111/j.1749-6632.1990.tb16904.x 2252328

[B149] Michael E. (1961). Overtraining in Athletics. The J. Sports Med. Phys. Fitness 1, 97–98.

[B150] Morgan W. P. Brown D. R. Raglin J. S. O'Connor P. J. Ellickson K. A. (1987). Psychological Monitoring of Overtraining and Staleness. Br. J. Sports Med. 21 (3), 107–114. 10.1136/bjsm.21.3.107 3676635 PMC1478455

[B151] Morgan W. P. Costill D. L. Flynn M. G. Raglin J. S. O?Connor P. J. (1988). Mood Disturbance Following Increased Training in Swimmers. Med. Sci. Sports Exerc. 20 (4), 408–414. 10.1249/00005768-198808000-00014 3173050

[B152] Mosca A. Leclerc M. Hugot J. P. (2016). Gut Microbiota Diversity and Human Diseases: Should We Reintroduce Key Predators in Our Ecosystem. Front. Microbiol. 7, 455. 10.3389/fmicb.2016.00455 27065999 PMC4815357

[B153] Mountjoy M. (2014). The IOC Consensus Statement: beyond the Female Athlete Triad-Relative Energy Deficiency in Sport (RED-S). Br. J. Sports Med. 48 (7), 491–497. 10.1136/bjsports-2014-093502 24620037

[B154] Moylan S. Berk M. Dean O. M. Samuni Y. Williams L. J. O’Neil A. (2014). Oxidative & Nitrosative Stress in Depression: Why So Much Stress? Neurosci. Biobehavioral Rev. 45, 46–62. 10.1016/j.neubiorev.2014.05.007 24858007

[B155] Nedic Erjavec G. Sagud M. Nikolac Perkovic M. Svob Strac D. Konjevod M. Tudor L. (2021). Depression: Biological Markers and Treatment. Prog. Neuro-Psychopharmacology Biol. Psychiatry 105, 110139. 10.1016/j.pnpbp.2020.110139 33068682

[B156] O'Sullivan O. Cronin O. Clarke S. F. Murphy E. F. Molloy M. G. Shanahan F. (2015). Exercise and the Microbiota. Gut Microbes 6 (2), 131–136. 10.1080/19490976.2015.1011875 25800089 PMC4615660

[B157] Ortiz N. Hernandez R. D. Jimenez R. Mauledeoux M. Aviles O. (2018). Survey of Biometric Pattern Recognition via Machine Learning Techniques. ces 11 (34), 1677–1694. 10.12988/ces.2018.84166

[B158] Owen J. R. (1964). Problems in Training Oarsmen - Staleness. Bull. - Br. Assoc. Sport Med. 1 (1), 6. 10.1136/bjsm.1.1.6

[B159] Pareja-Galeano H. Sanchis-Gomar F. García-Giménez J. L. (2014). Physical Exercise and Epigenetic Modulation: Elucidating Intricate Mechanisms. Sports Med. 44 (4), 429–436. 10.1007/s40279-013-0138-6 24399634

[B160] Park C. Rosenblat J. D. Pan Z. Lee Y. Cao B. (2019). Stress, Epigenetics and Depression: A Systematic Review. Neurosci. Biobehav Rev. 102, 139–152. 10.1016/j.neubiorev.2019.04.010 31005627

[B161] Parmenter D. (1923). Some Medical Aspects of the Training of College Athletes. Boston Med. Surg. J. 189 (2), 45–50. 10.1056/nejm192307121890201

[B162] Parry-Billings M. Blomstrand E. McAndrew N. (1990). A Communicational Link between Skeletal Muscle, Brain, and Cells of the Immune System. Int. J. Sports Med. 11 (Suppl. 2), S122–S128. 10.1055/s-2007-1024863 2193890

[B163] Pope C. C. Penney D. Smith T. B. (2018). Overtraining and the Complexities of Coaches’ Decision-Making: Managing Elite Athletes on the Training Cusp. Reflective Pract. 19 (2), 145–166. 10.1080/14623943.2017.1361923

[B164] Posada-Quintero H. F. Reljin N. Moutran A. Georgopalis D. Lee E. C. H. Giersch G. E. (2019). Mild Dehydration Identification Using Machine Learning to Assess Autonomic Responses to Cognitive Stress. Nutrients 12 (1), 1–12. 10.3390/nu12010042 31877912 PMC7019291

[B165] Prokop D. (1952). Probleme des ȕbertrainings. Proc. Int. Conf. Sport Health, 73.

[B166] Raglin J. S. Morgan W. P. (1994). Development of a Scale for Use in Monitoring Training-Induced Distress in Athletes. Int. J. Sports Med. 15 (2), 84–88. 10.1055/s-2007-1021025 8157374

[B167] Ramson R. (2012). The Effect of 4-week Training Period on Plasma Neuropeptide Y, Leptin and Ghrelin Responses in Male Rowers. Eur. J. Appl. Physiol. 112 (5), 1873–1880. 10.1007/s00421-011-2166-y 21922260

[B168] Rasmussen M. Zierath J. R. Barrès R. (2014). Dynamic Epigenetic Responses to Muscle Contraction. Drug Discov. Today 19 (7), 1010–1014. 10.1016/j.drudis.2014.03.003 24631681

[B169] Reichenberg A. Yirmia R. Schuld A. Kraus T. (2001). Cytokine-associated Emotional and Cognitive Disturbances in Humans. Arch. Gen. Psychiatry 58 (5), 445–452. 10.1001/archpsyc.58.5.445 11343523

[B170] Ridaura V. K. Faith J. J. Rey F. E. Cheng J. Duncan A. E. Kau A. L. (2013). Gut Microbiota from Twins Discordant for Obesity Modulate Metabolism in Mice. Science 341 (6150), 1241214. 10.1126/science.1241214 24009397 PMC3829625

[B171] Ritchie M. D. Holzinger E. R. Li R. Pendergrass S. A. Kim D. (2015). Methods of Integrating Data to Uncover Genotype-Phenotype Interactions. Nat. Rev. Genet. 16 (2), 85–97. 10.1038/nrg3868 25582081

[B172] Robinson J. L. Nielsen J. (2016). Integrative Analysis of Human Omics Data Using Biomolecular Networks. Mol. Biosyst. 12 (10), 2953–2964. 10.1039/c6mb00476h 27510223

[B173] Saghiv M. S. Sagiv M. S. (2020). “Epigenetics in Exercise,” in Basic Exercise Physiology (Cham, Switzerland: Springer, 521–539. 10.1007/978-3-030-48806-2_12

[B174] Salonen A. de Vos W. M. (2014). Impact of Diet on Human Intestinal Microbiota and Health. Annu. Rev. Food Sci. Technol. 5, 239–262. 10.1146/annurev-food-030212-182554 24387608

[B175] Schatzberg A. F. (1998). Noradrenergic versus Serotonergic Antidepressants: Predictors of Treatment Response. J. Clin. Psychiatry 59 Suppl 14 (Suppl. 14), 15–18. 10.4088/jcp.v59n1007b 9818626

[B176] Schmidt T. S. B. Raes J. Bork P. (2018). The Human Gut Microbiome: From Association to Modulation. Cell 172 (6), 1198–1215. 10.1016/j.cell.2018.02.044 29522742

[B177] Schwartz T. S. (2020). The Promises and the Challenges of Integrating Multi-Omics and Systems Biology in Comparative Stress Biology. Integr. Comp. Biol. 60 (1), 89–97. 10.1093/icb/icaa026 32386307

[B178] Sejnowski T. J. Churchland P. S. Movshon J. A. (2014). Putting Big Data to Good Use in Neuroscience. Nat. Neurosci. 17 (11), 1440–1441. 10.1038/nn.3839 25349909 PMC4224030

[B179] Selye H. (1976). The Stress of Life. New York: McGraw-Hill.

[B181] Sharples A. P. Stewart C. E. Seaborne R. A. (2016). Does Skeletal Muscle Have an 'epi'‐memory? the Role of Epigenetics in Nutritional Programming, Metabolic Disease, Aging and Exercise. Aging Cell 15 (4), 603–616. 10.1111/acel.12486 27102569 PMC4933662

[B182] Siever L. J. Coccaro E. F. Benjamin E. Davis K. L. (1986). Adrenergic and Serotonergic Receptor Responsiveness in Depression. Ciba Found. Symp. 123, 148–163. 10.1002/9780470513361.ch9 3816411

[B183] Silva F. C. D. (2020). Effects of Physical Exercise on the Expression of MicroRNAs: A Systematic Review. J. Strength Cond Res. 34 (1), 270–280. 10.1519/jsc.0000000000003103 31877120

[B184] Slyepchenko A. Maes M. Jacka F. N. Köhler C. A. Barichello T. McIntyre R. S. (2017). Gut Microbiota, Bacterial Translocation, and Interactions with Diet: Pathophysiological Links between Major Depressive Disorder and Non-communicable Medical Comorbidities. Psychother Psychosom 86 (1), 31–46. 10.1159/000448957 27884012

[B185] Smith L. L. (2000). Cytokine Hypothesis of Overtraining: a Physiological Adaptation to Excessive Stress? Med. Sci. Sports Exerc. 32 (2), 317–331. 10.1097/00005768-200002000-00011 10694113

[B186] Snyder A. C. Jeukendrup A. E. Hesselink M. K. Kuipers H. Foster C. (1993). A Physiological/psychological Indicator of Over-reaching during Intensive Training. Int. J. Sports Med. 14 (1), 29–32. 10.1055/s-2007-1021141 8440542

[B187] Snyder A. C. (1995). Overtraining Following Intensified Training with normal Muscle Glycogen. Med. Sci. Sports Exerc. 27 (7), 1063–1070. 10.1249/00005768-199507000-00016 7564974

[B188] Solomon M. B. Furay A. R. Jones K. Packard A. E. B. Packard B. A. Wulsin A. C. (2012). Deletion of Forebrain Glucocorticoid Receptors Impairs Neuroendocrine Stress Responses and Induces Depression-like Behavior in Males but Not Females. Neuroscience 203, 135–143. 10.1016/j.neuroscience.2011.12.014 22206943 PMC4000316

[B189] Sporns O. Chiavlo D. R. Kaiser M. Hiletag C. C. (2004). Organization, Development and Function of Complex Brain Networks. Trends Cogn. Sci. 8 (9), 418–425. 10.1016/j.tics.2004.07.008 15350243

[B190] Steinacker J. r. M. Lormes W. Reissnecker S. Liu Y. (2004). New Aspects of the Hormone and Cytokine Response to Training. Eur. J. Appl. Physiol. 91 (4), 382–391. 10.1007/s00421-003-0960-x 14608461

[B191] Stellingwerff T. (2021). Overtraining Syndrome (OTS) and Relative Energy Deficiency in Sport (RED-S): Shared Pathways, Symptoms and Complexities. Sports Med. 51 (11), 2251–2280. 10.1007/s40279-021-01491-0 34181189

[B192] Stone M. H. (1991). Overtraining: A Review of the Signs, Symptoms and Possible Causes. J. Strength Conditioning Res. 5 (1), 35–50. 10.1519/00124278-199102000-00006

[B193] Sun Y. V. Hu Y.-J. (2016). Integrative Analysis of Multi-Omics Data for Discovery and Functional Studies of Complex Human Diseases. Adv. Genet. 93, 147–190. 10.1016/bs.adgen.2015.11.004 26915271 PMC5742494

[B194] Tanaka H. West K. A. Duncan G. E. Bassett D. R. (1997). Changes in Plasma Tryptophan/branched Chain Amino Acid Ratio in Responses to Training Volume Variation. Int. J. Sports Med. 18 (4), 270–275. 10.1055/s-2007-972632 9231843

[B195] Terjung R. (1979). Endocrine Response to Exercise. Exerc. Sport Sci. Rev. 7, 153–180. 10.1249/00003677-197900070-00007 399464

[B196] Ticinesi A. Lauretani F. Tana C. Nouvenne A. Ridolo E. Meschi T. (2019). Exercise and Immune System as Modulators of Intestinal Microbiome: Implications for the Gut-Muscle axis Hypothesis. Exerc. Immunol. Rev. 25, 84–95. 30753131

[B197] Turnbaugh P. J. Ridaura V. K. Faith J. J. Rey F. E. Knight R. Gordon J. I. (2009). The Effect of Diet on the Human Gut Microbiome: a Metagenomic Analysis in Humanized Gnotobiotic Mice. Sci. Transl Med. 1 (6), 6ra14. 10.1126/scitranslmed.3000322 PMC2894525 20368178

[B198] Turnbaugh P. J. Ley R. E. Hamady M. Fraser-Liggett C. M. Knight R. Gordon J. I. (2007). The Human Microbiome Project. Nature 449 (7164), 804–810. 10.1038/nature06244 17943116 PMC3709439

[B199] Urhausen A. Gabriel H. H. W. Kindermann W. (1998). Impaired Pituitary Hormonal Response to Exhaustive Exercise in Overtrained Endurance Athletes. Med. Sci. Sports Exerc. 30 (3), 407–414. 10.1097/00005768-199803000-00011 9526887

[B200] Urhausen A. Gabriel H. Kindermann W. (1995). Blood Hormones as Markers of Training Stress and Overtraining. Sports Med. 20 (4), 251–276. 10.2165/00007256-199520040-00004 8584849

[B201] Urhausen A. Kindermann W. (2002). Diagnosis of Overtraining. Sports Med. 32 (2), 95–102. 10.2165/00007256-200232020-00002 11817995

[B180] US Department of Health and Human Services (1993). Depression Is a Treatable Illness. Rockville, MD: Department of Health and Human Services, 1–33.

[B202] Uusitalo A. L. T. Huttunen P. Hanin Y. Uusitalo A. J. Rusko H. K. (1998). Hormonal Responses to Endurance Training and Overtraining in Female Athletes. Clin. J. Sport Med. 8 (3), 178–186. 10.1097/00042752-199807000-00004 9762476

[B203] van Borselen F. (1992). EXERCISE PHYSIOLOGY CORNER: The Role of Anaerobic Exercise in Overtraining. Strength Conditioning J. 14 (3), 49. 10.1519/0744-0049(1992)014<0074:troaei>2.3.co;2

[B204] van Eeden W. A. Hemret A. M. V. Carlier I. V. E. Pennnix B. W. J. H. (2020). Basal and LPS-Stimulated Inflammatory Markers and the Course of Individual Symptoms of Depression. Transl Psychiatry 10 (1), 235. 10.1038/s41398-020-00920-4 32669537 PMC7363825

[B205] Verma S. K. Mahindroo S. R. Kansal D. K. (1978). Effect of Four Weeks of Hard Physical Training on Certain Physiological and Morphological Parameters of Basket-ball Players. J. Sports Med. Phys. Fitness 18 (4), 379–384. 745386

[B206] Von Bertalanffy L. (1950). The Theory of Open Systems in Physics and Biology. Science 111 (2872), 23–29. 10.1126/science.111.2872.23 15398815

[B207] Walsh L. L. (1980). Differences in Food, Water, and Food-Deprivation Water Intake in 16 Strains of Rats. J. Comp. Physiol. Psychol. 94 (4), 775–781. 10.1037/h0077698

[B208] Widmann M. Niess A. M. Munz B. (2019). Physical Exercise and Epigenetic Modifications in Skeletal Muscle. Sports Med. 49 (4), 509–523. 10.1007/s40279-019-01070-4 30778851

[B209] Wilmanski T. Diener C. Rappaport N. Patwardhan S. Wiedrick J. Lapidus J. (2021). Gut Microbiome Pattern Reflects Healthy Ageing and Predicts Survival in Humans. Nat. Metab. 3 (2), 274–286. 10.1038/s42255-021-00348-0 33619379 PMC8169080

[B210] Wischnia B. (1994b). “A Time to Reflect,” in Runners World (Mountain View, CA: World Publications), 80–84.

[B211] Wischnia B. (1994a). “Comeback at Comrades,” in Runners World, 76–77.

[B212] Wittert G. A. (1996). Adaptation of the Hypothalamopituitary Adrenal axis to Chronic Exercise Stress in Humans. Med. Sci. Sports Exerc. 28 (8), 1015–1019. 10.1097/00005768-199608000-00011 8871911

[B213] Wolf J. (1957). Ein beitrag zur frage des ȕbertrainings. Sportmedizin 2, 33–38.

[B214] Wu G. D. Chen J. Hoffmann C. Bittinger K. Chen Y.-Y. Keilbaugh S. A. (2011). Linking Long-Term Dietary Patterns with Gut Microbial Enterotypes. Science 334 (6052), 105–108. 10.1126/science.1208344 21885731 PMC3368382

[B215] Yao H. Zhang N. Zhang R. Duan M. Xie T. Pan J. (2020). Severity Detection for the Coronavirus Disease 2019 (COVID-19) Patients Using a Machine Learning Model Based on the Blood and Urine Tests. Front. Cel Dev. Biol. 8, 683. 10.3389/fcell.2020.00683 PMC7411005 32850809

[B216] Yuan X. Xu S. Huang H. Liang J. Wu Y. (2018). Influence of Excessive Exercise on Immunity, Metabolism, and Gut Microbial Diversity in an Overtraining Mice Model. Scand. J. Med. Sci. Sports 28 (5), 1541–1551. 10.1111/sms.13060 29364545

[B217] Yugi K. Kubota H. Hatano A. Kuroda S. (2016). Trans-Omics: How to Reconstruct Biochemical Networks across Multiple 'Omic' Layers. Trends Biotechnol. 34 (4), 276–290. 10.1016/j.tibtech.2015.12.013 26806111

[B218] Zautra A. J. Reich J. W. (1983). Life Events and Perceptions of Life Quality: Developments in a Two-Factor Approach. J. Community Psychol. 11 (2), 121–132. 10.1002/1520-6629(198304)11:2<121:aid-jcop2290110206>3.0.co;2-v

[B219] Zunszain P. A. Anacker C. Cattaneo A. Carvalho L. A. Pariante C. M. (2011). Glucocorticoids, Cytokines and Brain Abnormalities in Depression. Prog. Neuro-Psychopharmacology Biol. Psychiatry 35 (3), 722–729. 10.1016/j.pnpbp.2010.04.011 PMC3513408 20406665

