# Supplementary material for: Overtraining Syndrome as a Complex Systems Phenomenon
Source: Front Netw Physiol. 2022 Jan 18;1:794392. doi: 10.3389/fnetp.2021.794392 (PMC10013019; doi:10.3389/fnetp.2021.794392)
Supplement: Supplementary file 1 [file Table1.docx]

**Supplemental Table 1. Summaries of selected publications (1923 - 2018) that illustrate the evolution of information and concepts regarding overtraining/staleness. These published observations may or may not reflect present-day consensus (Meeusen, Duclos et al. 2013) regarding the Overtraining Syndrome (OTS).**

| **Year** | **Sport and description of characteristics** | **References** |
| --- | --- | --- |
| 1923- 1928 | A syndrome of reduced athletic performance was first described in a medical journal by Parmenter in 1923. Brustmann & Hoske (Brustmann and Hoske 1928) designated this syndrome overtraining in 1928. | (Parmenter 1923, Brustmann and Hoske 1928)^a^ |
| 1933-1957 | Anecdotal accounts and systematic descriptions of athlete complaints experienced by overtrained athletes were published in journals and conference proceedings. | (Knoll and Arnold 1933, Kereszty 1947, Prokop 1952, Wolf 1957)^a^ |
| 1955 | Staleness (decreased athletic performance) due to overtraining or overworking was observed among experienced collegiate athletes and teams. Physiological and psychological staleness were viewed as separate entities. The former was associated with inadequate rest, inadequate sleep, improper diet, and predisposition to illness. The latter involved boredom or loss of interest, drive, zest for training and competition. Some athletes appeared to be nervous (e.g., jumpy) about their training or expressed a desire for the season to end^b^. | (Counsilman 1955) |
| 1961 | Collegiate runners experienced increased sensitivity to stress (i.e., apparent increased adrenal gland sensitivity, and over-stimulation). They required rest and medical care. Adrenal dysfunction was suspected. Proposed etiology: combined emotional, physical, and environmental stress with little chance for recovery. | (Michael 1961) |
| 1964 | Staleness was identified among amateur oarsmen (rowers) when a crew team or an individual failed to reproduce in competition the abilities shown in training and practice. Staleness was believed to be psychological in origin with physical manifestations such as lethargy, lack of interest, tiredness, insomnia, and lack of will or even desire to win. Less obvious signs included abnormal anxiety about winning, increasing irritability about unimportant matters, and complaints of trifling ailments.^b^ The author noted that staleness cannot be rectified easily. Thus, prevention is essential and warning signs should be monitored. | (Owen 1964) |
| 1971 | Subjective and objective signs of overtraining were presented. The latter included performance decline, longer recovery periods, weight loss, increased heart rate at rest or during exercise, and increased muscle injuries. The authors also described (a) stressful personal and environmental factors which add to the stress of physical training, (b) preventive measures, and (c) recommendations for altering the training regimen to encourage recovery. | (Mellerowicz and Barron 1971) |
| 1982 | This article was one of the first to use the term Overtraining Syndrome in its title. Emotional and physical warning signs were described, as experienced during weight training, distance running, and interval training. When warning signs are observed, recovery should be encouraged by manipulating the intensity, frequency and duration of training on an individual basis. | (Fleck and Kraemer 1982) |
| 1985 | A research study involved both overtrained and asymptomatic runners. The overtrained athletes presented with impaired training and racing times, apathy, and sensations of heavy legs; hormonal responses suggested neuroendocrine dysfunction. | (Barron, Noakes et al. 1985) |
| 1987 | Staleness was characterized by chronic fatigue, depressed appetite, weight loss, insomnia, decreased libido, muscle soreness and elevated depression or tension.^b^ Monitoring of mood states was recommended as a potential method of quantifying distress and titrating each athlete’s training loads and/or rest. | (Morgan, Brown et al. 1987) |
| 1988 | This controlled field study focused on the psychological/mood responses of 12 highly-trained collegiate swimmers before, during and after 10 d of increased training. Athletes were monitored each morning using the Profile of Mood States questionnaire (POMS), a muscle soreness scale, and a 24-h diary (i.e., evaluating general well-being, illnesses, and sleep patterns). Significant increases were observed in ratings of depression, anger, fatigue, and the composite measure of mood (i.e. mood profile); sleep patterns were unchanged. These significant mood changes, as well as ratings of muscle soreness and general sense of well-bring, plateaued by day 5 of increased training. However, the authors concluded that this acute 10 d period of overreaching does not mean that the staleness syndrome can necessarily be produced within a short, intense training period. Physiological responses are discussed below. | (Morgan, Costill et al. 1988) |
| 1988 | This study involved physiological responses of the same 12 collegiate swimmers described in the previous item. They had been training intensely for 6 months before their average training distance in the pool increased from 4,266 to 8,970 m per day (2 sessions/d at 94% VO_2max_); this resulted in difficulty completing training sessions and the mood changes described above (Morgan, Costill et al. 1988). However, their tethered swimming power output (laboratory), in-pool sprint speed, endurance performance, and aerobic capacity were unchanged by 10 d of overreaching. Across the 10 d of increased training, posterior deltoid muscle biopsy analysis indicated that aerobic enzyme (citrate synthase) concentration was unchanged but muscle glycogen concentration decreased. These results suggested that some swimmers may experience chronic muscular fatigue owing to a failure to ingest sufficient carbohydrate to match the demands of heavy training.^c^ | (Costill, Flynn et al. 1988) |
| 1988 | Overreaching, overtraining, staleness, and OTS were distinguished and defined. Possible mechanisms of OTS were described. Evidence suggested that nonfunctional overreaching and OTS (see Figure 1) result from a hypothalamic dysfunction, not malfunction of peripheral hormonal organs. | (Kuipers and Keizer 1988) |
| 1990 | Laboratory testing of 12 underperforming elite athletes (i.e., their status was not designated as either OTS or overreaching) was conducted. Following 3-5 weeks of rest, measurements indicated (a) significantly increased (p<0.05) body weight, maximal respiratory exchange ratio, maximal oxygen consumption, and heart rate at the anaerobic threshold, and (b) significantly reduced rating of fatigue and increased vigor. | (Koutedakis, Budgett et al. 1990) |
| 1991 | An extensive list was presented of signs and symptoms that previously had been associated with the process of overtraining. This list included 40 physiological/performance factors, 12 psychological/information processing characteristics, 14 immunological variables, and 18 biochemical analytes. | (Fry, Morton et al. 1991) |
| 1992 | Because the search for variables that can detect early overtraining was ongoing, this investigation combined laboratory and field testing of 7 male competitive cyclists who intensified their normal training for 2 wk (IT), to achieve a state of short-term overtraining. Before, during and after IT, plus 2 wk of recovery, observations included cycle ergometry testing, an outdoor 8.5 km time trial, measurements of reaction time and visual perception, and a questionnaire. At the end of IT, these variables declined: time trial performance, maximal cycle ergometry power output, maximal heart rate, maximal oxygen consumption. After 2 wk of recovery, cyclists reached a state of supercompensation and all variables returned to their reference level or showed an improvement. However, indicative of over-reaching, participants still reported feeling tired. | (Jeukendrup, Hesselink et al. 1992) |
| 1993 | The process of overtraining causes imbalances between training and recovery, exercise and exercise capacity, stress and stress tolerance. The authors observed that symptomatology changed during the 50 years prior to this publication, from excitation and restlessness (i.e., sympathetic nervous system predominance) to phlegmatic behavior and inhibition (parasympathetic predominance). They attributed this change to an increased volume of training at high exercise intensities. | (Lehmann, Foster et al. 1993) |
| 1995 | A controlled field study monitored 14 elite male and female swimmers at 5 points during a 6-month season, including tapering of training and a major culminating competition. Staleness scores were calculated for each athlete using (a) performance change from early- to late-season and (b) daily fatigue ratings. Subjective ratings of well-being predicted improvement of competitive performance. In this small athlete sample, 21% were diagnosed as stale during a single 6-month season. The end-of-season taper did not provide these stale swimmers with adequate time to recover completely prior to competition; their fatigue and muscle soreness ratings were significantly greater than for nonstale swimmers. | (Hooper, Mackinnon et al. 1995) |
| 1997 | This review of endurance sports considered the question, Where is the borderline between overreaching and long-term overtraining? Summarizing research observations of swimmers, soldiers, cyclists, joggers, and elite rowers, the authors concluded that an increased risk of staleness may be assumed to occur at about 3 weeks of intensified or prolonged monotonous endurance training. | (Lehmann, Lormes et al. 1997) |
| 1998 | OTS was defined as a condition of fatigue and underperformance, often associated with frequent infections and depression, which occurs following hard training and competition. Sleep disturbances exist in over 90% of OTS cases, appearing as difficulty in getting to sleep, nightmares, waking in the night, and waking unrefreshed. Athletes may experience appetite changes, weight loss, loss of competitive drive and libido, anxiety, and irritability. An increased resting pulse rate, excessive sweating, or recurring upper respiratory tract infections may occur. Diagnosis of OTS is difficult because clinical signs often are caused by associated illness and are inconsistent. | (Budgett 1998) |
| 2000 | This insightful publication reviewed 7 hypothetical OTS mechanisms that have been advanced by researchers. Some have focused on the hypothalamus, which activates the autonomic nervous system, pituitary, and adrenal glands or gonads; this results in altered blood catecholamines (e.g., norepinephrine, epinephrine), cortisol, and testosterone levels. Other hypotheses focused on circulating amino acids (e.g., glutamine, tryptophan), muscle and liver glycogen depletion, and psychological monotony effects on physiological performance. The purpose of this paper is to integrate information pertaining to OTS into one paradigm, referred to as the cytokine hypothesis of overtraining (details appear in Table 1). An extensive list presents 84 distinct factors that have been associated with OTS, as originally published in 1991 (Fry, Morton et al. 1991). | (Smith 2000) |
| 2002 | Despite identification of several hypothetical mechanisms for OTS, its nature and diagnostic biomarkers remain elusive. Complicating description and recognition of OTS, considerable variability of signs and symptoms exists among individuals, across different sports, and between training regimens that emphasize intensity versus volume. This review article proposed a novel paradigm, based on OTS and clinical depression (i.e., major depression) involving remarkably similar signs and symptoms, brain structures, neurotransmitters, endocrine pathways and immune responses. This suggests similar etiologies for depression and OTS. Subsections of this article considered the complex nature of both conditions in terms of: neuroendocrine and immune responses, stress hormones, mood disturbances, neurotransmitters, regulation of membrane receptors, autonomic nervous system balance, and synaptic plasticity. | (Armstrong and VanHeest 2002) |
| 2009 | A 22-item training distress questionnaire was developed to monitor self-reported measures of training overload. The conceptual model combined measures of mood disturbance, perceived stress, and symptom intensity. It included six factors: depressed mood, perceived vigor, physical symptoms, sleep disturbance, perceived stress, general fatigue. Athletes (n = 492) completed the 10-item Perceived Stress Scale, the 24-item Brunel Mood Scale, and a checklist of 19 symptoms associated with acute overtraining. Comparisons of group means of these factors with a previously validated inventory were consistent with theoretical predictions, providing evidence of construct validity. Internal consistency of the subscales also was confirmed, with Cronbach alpha statistics ranging from 0.72 to 0.86. Data suggested that this conceptual model provided a sound conceptual basis for the assessment of training overload. | (Main and Grove 2009) |
| 2010 | Data from this research investigation of athletes (5 diagnosed with nonfunctional overreaching and 5 with OTS), when considered in light of previous studies (see (Budgett 1998) above), suggested that both conditions are neuroendocrine disorders. Specifically, hypothalamic dysfunction is more likely than malfunction of peripheral endocrine organs because these two exercise training syndromes have characteristics in common with other intensive and chronic stress disorders (e.g., depression, post-traumatic stress disorder). | (Meeusen, Nederhof et al. 2010) |
| 2010 | Viewing adaptation to systematic physical training as a psychobiological phenomenon which involves interactions of complex biological systems, this pioneering publication described how exercise-induced phenomena at different time scales and levels of biological system organization strongly indicate nonlinear integrative mechanisms at work. These mechanisms involve the dynamics of a variety of context dependent, task-specific, flexibly-assembled, collective factors. In addition to overtraining, the authors considered low workload density, delayed training effects, and exhaustion-induced exercise termination. The authors note that a small change in training history may produce an overtraining effect, and that no one-to-one relationship exists between the training workload stimulus and training effects. | (Hristovski, Venskaityte et al. 2010) |
| 2013 | This expert consensus statement was endorsed by the European College of Sport Science and the American College of Sports Medicine. The authors of the present review article consider this to be the authoritative document regarding the prevention, diagnosis, and treatment of OTS. Key concepts appear in **Supplemental Table 2**. | (Meeusen, Duclos et al. 2013) |
| 2017 | A systematic literature search was conducted to evaluate the roles that hormones play in OTS, overreaching, overtraining, and underperforming. The initial search yielded 835 articles which provided an overview of methodologies between the years 1985 – 2015. Athletes participated in 17 different sport/competitive activities. Among the 38 selected studies, 21 were performed in healthy athletes in whom nonfunctional overreaching (NO; see Figure 1) or functional overreaching (FO) was induced, whereas 17 were conducted in previously affected athletes (3 with and 14 without an overload training program); 26 included a control group of healthy athletes and 12 compared data to previous/basal levels of the same athletes. The remarkable size and breadth of studies posed the following limitations to understanding OTS and overreaching: the wide variety of research methods and sports, a lack of standardized criteria to differentiate OTS from FO and NO; a small number of participants in most studies, and few studies involving resistance training. Further, because many studies did not compare athletes with OTS to sedentary controls or trained athletes, altered hormone concentrations may not have distinguished training-induced physiological adaptions from reduced exercise performance. | (Cadegiani and Kater 2017) |
| 2018 | A qualitative research study involved 3 highly successful professional elite rowing coaches (i.e., of Olympic and world class athletes) as they predicted and managed the risk of overtraining during crucial periods of intense training. Three semi-structured, in-depth interviews were conducted with each coach, addressing (i) how they defined overtraining, (ii) why they considered overtraining important, and (iii) the cues they used to determine whether a rower was at risk of impending overtraining. Analysis of interviews indicated that coaches knew that pushing the intensity and duration of training carries a risk of overtraining, but this approach was viewed as necessary to achieve the highest level of international success. These coaches attempted to improve performance fractions of a percent but realized that the process represented a fine line between intense, prolonged training and overtraining. Interestingly, there were no straightforward rules or measurement tools that informed their decisions. Coaches relied on changes in performance, observation, communication, and knowledge of each rower. Their decision-making was highly intuitive, weighing the risks versus benefits of maintaining or reducing the training overload. | (Pope, Penney et al. 2018) |

^a^, cited and described in reference (Kereszty 1971)^b^, the classic book *The Stress of Life* (Selye 1976) described the following signs and symptoms of stress that could be experienced during daily activities: irritability, hyper-excitation or depression, inability to concentrate, loss of the joyful enjoyment of living, being keyed up , insomnia, frequent need to urinate, diarrhea, indigestion, stomach queasiness, tendency to be easily startled, excessive appetite or loss of appetite, accident proneness, nightmares; ^c^, two distinctions are warranted. First, swimmers did not meet contemporary criteria of OTS (i.e., performance did not decline) and investigators did not claim that OTS occurred. Second, this investigation is unique because it presents a nutritional aspect of overreaching training (e.g., regarding daily carbohydrate intake) that may influence interpretation of experimental data as well as athlete sensations of muscular fatigue. See section 4.5 above titled, Diet, Macronutrients, and Energy Availability
