# Supplementary figures and images for: Overtraining Syndrome as a Complex Systems Phenomenon
Source: Front Netw Physiol. 2022 Jan 18;1:794392. doi: 10.3389/fnetp.2021.794392 (PMC10013019; doi:10.3389/fnetp.2021.794392)

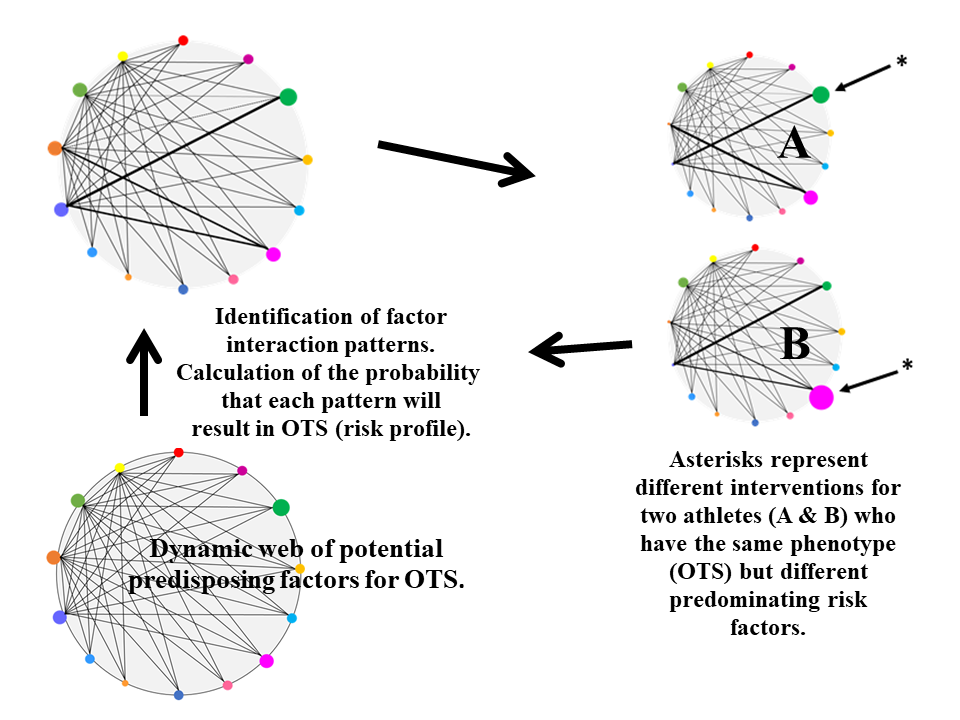

Supplement: Supplementary file 2 [file Figure4.TIF]
