# Supplementary material for: Overtraining Syndrome as a Complex Systems Phenomenon
Source: Front Netw Physiol. 2022 Jan 18;1:794392. doi: 10.3389/fnetp.2021.794392 (PMC10013019; doi:10.3389/fnetp.2021.794392)
Supplement: Supplementary file 3 [file Table2.docx]

**Supplemental Table 2. Selected publications that associated the overtraining syndrome or nonfunctional overreaching with neuroendocrine responses to stress.**

| **Year** | **Description of Findings/Conclusions** | **Reference** |
| --- | --- | --- |
| 1985 | Hypothalamic-pituitary function was studied in 5 asymptomatic and 4 overtrained^a^ male marathon runners (i.e., evaluated when they were overtrained and after 4 weeks of rest). Insulin-induced hormone responses (plasma cortisol, adrenocorticotrophic hormone, growth hormone, prolactin, luteinizing hormone, thyroid stimulating hormone) in the overtrained athletes were lower than their own responses after 4 weeks of rest, and lower than the asymptomatic runners. These results suggested hypothalamic but not pituitary dysfunction. | (Barron, Noakes et al. 1985) |
| 1988 | This review paper distinguished overreaching from overtraining.^a^ Describing overtraining and its clinical manifestations as a stress response, possible causes include: an imbalance of excitation and inhibition of the neuroendocrine system, hypothalamic dysfunction (see Barron et al., 1985 above), increased cortisol release from the adrenal glands, downregulation of cortisol receptors in the brain, a transient loss of pituitary responsiveness, impairment of negative feedback that terminates the stress response, altered balance between catabolic and anabolic hormones, and exhaustion of the pituitary-adrenal-gonadal axis. The specific neuroendocrine mechanisms involved in overtraining are unknown. | (Kuipers and Keizer 1988) |
| 1991 | This study exposed experienced 8 middle- and long-distance runners to 4 weeks of progressively increasing training volume (e.g., from 85.9 to 174.6 km/wk, 6 d/wk).^d^ Plasma and urinary cortisol, catecholamines (noradrenaline, adrenaline, dopamine), cardiovascular, and metabolic variables were examined. Endurance capacity and/or treadmill performance decreased in all athletes at the end of the 4-wk observation period. Several plasma constituents decreased, whereas others remained constant. Importantly, the authors concluded that increased plasma noradrenaline concentration during treadmill running, and markedly reduced basal 24-h urinary catecholamine and cortisol excretion, were relevant to the diagnosis of short-term overtraining.^b^ | (Lehmann, Dickhuth et al. 1991) |
| 1993 | This review article considered neuroendocrine responses due to short-term overtraining and long-term overtraining.^a^ The following factors were proposed as possible mechanisms: a change of target organ sensitivity to neurotransmitters such as norepinephrine and epinephrine, changes in sympathetic or parasympathetic nervous system effects, dysfunction of the adrenal glands, and plasma levels of unbound cortisol and testosterone as indicators of catabolic and anabolic tissue activity. However, at the time of this publication, the mosaic of findings did not permit a consensus of assessment or a mechanism. | (Lehmann, Foster et al. 1993) |
| 1993 | This controlled field study monitored 14 elite male and female swimmers at 5 points during a 6-month competitive season. Considering all athletes as a single group, plasma cortisol and norepinephrine levels were not different at any time point throughout the season; plasma epinephrine decreased significantly only at the post-season testing session. Three swimmers were determined to be overtrained^c^; their plasma norepinephrine concentrations trended higher than non-stale swimmers from mid-season onward, and were significantly higher (P<.001) during tapering which occurred prior to the season-culminating competition. | (Hooper, MacKinnon et al. 1993) |
| 1997 | This review of endurance sports provides extensive coverage of hypothetical mechanisms that may underlie overtraining and overreaching^a^, including decreased intrinsic sympathetic activity (i.e., catecholamine excretion), reduced sensitivity of receptors to catecholamines (or reduced receptor density), and diminished sensitivity of adrenal glands to ACTH which affects cortisol release. | (Lehmann, Lormes et al. 1997) |
| 1997 | Resistance training overreaching and overtraining were the topics of this review paper. Depending on whether training volume or training intensity are excessive, resistance exercise can result in differential responses. The neuroendocrine responses to high volume resistance training appear to be similar to overtraining for aerobic activities, whereas excessive resistance training intensity produces a different neuroendocrine profile. Multiple anabolic and stress hormones were discussed. | (Fry and Kraemer 1997) |
| 1998 | The plasma stress hormones epinephrine and norepinephrine increase in overtrained athletes versus control subjects; cortisol may or may not increase. | (Budgett 1998) |
| 2002 | It is not widely recognized that overtraining syndrome (OTS)^a^ and clinical depression involve remarkably similar signs and symptoms. They also may involve similar brain structures, neurotransmitters, endocrine pathways and immune responses. In this review paper, the authors proposed that OTS and depression have similar etiologies. Several effects of the neurotransmitter serotonin are important in the etiology of both clinical depression and OTS, and the regulatory and integrating functions of the hypothalamus. Further, central serotonergic systems influence the regulation of hormones that are involved in the body’s responses to stress, and act upon the sympathetic nervous system and the hypothalamic-pituitary-adrenal axis (HPA). Clinically depressed patients exhibit dysfunction of the HPA axis, as do athletes who exhibit OTS. These numerous shared characteristics may offer insights into the mechanism of OTS and encourage testable experimental hypotheses. | (Armstrong and VanHeest 2002) |
| 2004 | Although the available scientific and anecdotal evidence supports the existence of OTS^c^ , there is no definitive diagnostic tool, inter-individual and between-study variabilities are large, and few well controlled studies exist. Most studies of intense training have investigated overreaching, not OTS. At the time of this publication, no evidence suggested that overreaching precedes OTS, that these conditions exist on a continuum, or that symptoms of OTS are more severe. | (Halson, Lancaster et al. 2004) |
| 2004 | This case-controlled study involved one athlete who had been clinically diagnosed with OTS; he complained about his inability to complete any training session during the last several months, needed a long recovery period, and participated in psychological counselling for 1 year. Seven trained cyclists were tested as a healthy comparison group, before (T) and after (OR) a 10-day training camp. The test protocol, which involved two bouts of maximal exercise, also was utilized in a later confirmatory publication; see (Meeusen, Nederhof et al. 2010) below. Blood was analyzed for growth hormone, cortisol, ACTH, and prolactin. The latter two hormone responses to the second exercise test were significantly different (i.e., the increase during T > OR > OTS); this was evidence of an altered and dysfunctional hypothalamic–pituitary axis response in the athlete with OTS. | (Meeusen, Piacentini et al. 2004) |
| 2004 | This review paper summarized the hormonal responses to exercise training and proposed a hypothetical biphasic model in which (a) acute training responses mainly involve peripheral muscular damage and metabolic needs, and (b) chronic training responses involve changes in tissue metabolism, somatic growth, body composition, organ function, and central regulatory disturbances. When a training overload is prolonged and intense, the adrenal glands become less responsive and there exists a depression of the HPA axis, resulting in a decrease of circulating levels of catecholamines. The authors noted that similar responses occur for all hypothalamic-pituitary-peripheral hormone axes. This overtrained state^a^ can be aggravated by limited recovery, substrate availability and protein synthesis, as well as the influences of other stressors. | (Steinacker, Lormes et al. 2004) |
| 2005 | Providing an extensive review of OTS research, potential biomarkers, and possible underlying mechanisms, this book chapter concludes that the causes of OTS^b^ are difficult to determine from previous research because some sports possess a combination of high-force and high-endurance characteristics, and because each sport is unique. Thus, previous publications report different physiological responses, adaptations, and maladaptations to sport-specific training stresses. | (Fry et al. 2005) |
| 2005 | This paper reviews the acute effects of resistance exercise and chronic effects of long-term resistance training on the following hormones: cortisol, testosterone, growth hormone, insulin-like growth factors, insulin, thyroid hormones, leptin, and estrogens. Overreaching and OTS are considered separately.^b^ Chronic adaptations of neuroendocrine function (i.e., including cortisol) appear to be minimal. Acute hormonal responses are related to the intensity and volume of the training session, genetic predisposition, sex, fitness level, nutrition, overreaching, OTS, and circadian patters of hormone secretion. | (Kraemer and Ratamess 2005) |
| 2006 | This controlled laboratory study examined performance and skeletal muscle tissue responses to high-intensity resistance exercise overtraining (daily for 2 wk). Weight-trained men were divided into overtrained (OT)^b,d^ and control groups. Biopsies from vastus lateralis muscle and nocturnal urinary epinephrine (U_EPI_) were assessed pre- and post-training; U_EPI_ reflected sympathetic nervous system activity; biopsies were analyzed for β_2_ adrenergic receptors (β2-AR). Circulating epinephrine binds to this receptor and activates numerous intracellular regulatory signaling pathways. Overtraining was verified by a decrease in strength and a 36.3% decrease in mean power output. A large increase in the U_EPI_:β_2_-AR ratio indicated that receptor sensitivity had decreased in the OT group but not the control group. | (Fry, Schilling et al. 2006) |
| 2007 | This review article focuses on the involvement of the central nervous system in OTS and fatigue. Research evidence suggests that the central neurotransmitter serotonin acts on the sympathetic nervous system and the HPA axis; reciprocally, glucocorticoids (i.e., cortisol) and catecholamines (epinephrine, norepinephrine, dopamine) affect central serotonergic systems. In pathological situations (i.e., major depression and possibly OTS), glucocorticoids and catecholamines apparently fail to restrain the HPA response to stress. Thus, neurotransmitters may play a key role in the maladaptations which characterize OTS. | (Meeusen, Watson et al. 2007) |
| 2010 | 10 underperforming athletes from 8 different sports performed 2 laboratory maximal exercise tests (24-25 min duration) to exhaustion with 4 h rest between. Based on the duration of recovery after testing, NFO was retrospectively diagnosed in 5 athletes (1- to 6-month recovery), and OTS was diagnosed in 5 other athletes (1 to >5 y recovery required).^c^ Venous blood was analyzed before and after both maximal exercise tests, to determine if specific variables could distinguish NFO from OTS. The authors concluded that NFO might be distinguished from OTS based on plasma ACTH and prolactin responses. In agreement with previous studies, these data suggested that NFO and OTS involve hypothalamic dysfunction rather than a malfunction of peripheral hormonal organs. | (Meeusen, Nederhof et al. 2010) |
| 2017 | A systematic literature search was conducted in the PUBMED, MEDLINE and Cochrane databases using 16 endocrine-related keywords, to evaluate the roles of hormones in OTS, functional overreaching (FO), and NFO (see Figure 1). The initial search yielded 835 articles, 38 of which were selected this analysis. There were three types of endocrine assessments of athletes: basal hormones in previously affected athletes, resting levels after overload training, and acute hormone responses to endocrine stimulation tests. The authors concluded that basal plasma concentrations are not useful as biomarkers of OTS or overreaching. Endurance sport athletes generally showed a reduction of catecholamine levels. | (Cadegiani and Kater 2017) |
| 2018 | This study aimed to compare the HPA axis responses of 14 overtrained athletes^c^ (OTS) to 25 healthy physically active subjects (PA) and 12 healthy non-active subjects (NA). This project measured plasma growth hormone (GH) and prolactin (PRL) responses to administration of intravenous insulin, a gold-standard and non-exercising endocrine stimulation test (ITT). Analyses determined that GH and PRL responses to the insulin tolerance test were blunted in OTS (versus the PA group). Unstimulated, resting basal concentrations of GH and PRL were lower in OTS (versus PA) but were similar to NA. In PA (compared to NA), growth hormone and prolactin responses to ITT were exacerbated. Based on their time-to-hypoglycemia during ITT, the authors recommended that athletes with OTS increase their dietary carbohydrate intake prior to training sessions to avoid hypoglycemia, because athletes with OTS may be more chronically and severely depleted of carbohydrates and glycogen. | (Cadegiani and Kater 2018b) |
| 2019 | The 51 subjects were identical to those in the preceding study (Cadegiani and Kater 2018b), identified as OTS, healthy physically active (PA), and healthy non-active (NA) men. A sequence of 3 gold standard non-exercising hormone tests were performed in all subjects, including the cosyntropin stimulation test, insulin tolerance test, and salivary cortisol rhythm test; these validated tests are endorsed by endocrine societies. Test results indicated that healthy PA experienced hormonal conditioning adaptations (versus healthy NA), but those with OTS exhibited a blunted optimal hormone response to non-exercise stress (i.e., located in the hypothalamus and/or pituitary but not the adrenal glands); similar to deconditioning, this may explain the decreased performance and reduced time-to-fatigue observed during OTS. | (Cadegiani and Kater 2019a) |
| 2019 | This investigation was designed to simplify and optimize the distinction between NFO and OTS. Test subjects (n = 100, 74 males), without (i.e., healthy controls) and with complaints of underperformance and fatigue, performed 2 laboratory maximal exercise tests to exhaustion with 4 h rest between (protocol described above, see (Meeusen, Nederhof et al. 2010)(28)). Before and after both exercise tests, blood was sampled and analyzed for the hormones cortisol, ACTH, prolactin, and growth hormone. Time of day and meal consumption were controlled. Statistical discriminant analysis indicated that hormonal (ACTH and prolactin) and psychological (ratings of fatigue and tension) responses to the second exercise test provided an accurate diagnosis of NFO and OTS with 98% sensitivity. | (Buyse, Decroix et al. 2019) |

^a^, published before currently-accepted criteria and operational definitions for OTS and NFO were published in the joint consensus statement of the European College of Sport Science and American College of Sports Medicine (Meeusen, Duclos et al. 2013)^b^, overtraining was defined in terms of decreased performance; ^c^, overtraining was defined in terms of decreased performance plus other criteria (e.g., time to the complete recovery of performance, self-ratings of fatigue or mood, sleep quality); ^d^, overtraining was induced and verified by investigators as part of the experimental protocol; ^e^, neither overtraining nor OTS were operationally defined
