# Supplementary material for: Overtraining Syndrome as a Complex Systems Phenomenon
Source: Front Netw Physiol. 2022 Jan 18;1:794392. doi: 10.3389/fnetp.2021.794392 (PMC10013019; doi:10.3389/fnetp.2021.794392)
Supplement: Supplementary file 4 [file Table3.DOCX]

**Contribution to the field**

This review provides a comprehensive multi-domain rationale and guiding principles for researchers and clinicians to shift from existing paradigms, limited experimental studies to confirm or refute specific reductionist hypotheses, and single indicators to support diagnosis of overtraining syndrome (OTS) to the more revealing explanatory power of higher order and inclusive predictive models of the complex, dynamic, and integrated biological human systems, networks, and contributing factors underlying and that are fundamental to OTS.
